# Enclaves of genetic diversity resisted Inca impacts on population history

**DOI:** 10.1038/s41598-017-17728-w

**Published:** 2017-12-12

**Authors:** Chiara Barbieri, José R. Sandoval, Jairo Valqui, Aviva Shimelman, Stefan Ziemendorff, Roland Schröder, Maria Geppert, Lutz Roewer, Russell Gray, Mark Stoneking, Ricardo Fujita, Paul Heggarty

**Affiliations:** 1 Department of Linguistic and Cultural Evolution, Max Planck Institute for the Science of Human History, D-07745 Jena, Germany; 2 Centro de Investigación de Genética y Biología Molecular (CIGBM) Universidad de San Martín de Porres, Lima, Peru; 3 Departamento de Lingüística, Universidad Nacional Mayor de San Marcos, Lima, Peru; 4 Independent researcher, Chachapoyas (city), Chachapoyas, Peru; 5 Department of Evolutionary Genetics, Max Planck Institute for Evolutionary Anthropology, Leipzig, Germany; 6 Institute of Legal Medicine and Forensic Sciences, Department of Forensic Genetics, Charité—Universitätsmedizin Berlin, Berlin, Germany

## Abstract

The Inca Empire is claimed to have driven massive population movements in western South America, and to have spread Quechua, the most widely-spoken language family of the indigenous Americas. A test-case is the Chachapoyas region of northern Peru, reported as a focal point of Inca population displacements. Chachapoyas also spans the environmental, cultural and demographic divides between Amazonia and the Andes, and stands along the lowest-altitude corridor from the rainforest to the Pacific coast. Following a sampling strategy informed by linguistic data, we collected 119 samples, analysed for full mtDNA genomes and Y-chromosome STRs. We report a high indigenous component, which stands apart from the network of intense genetic exchange in the core central zone of Andean civilization, and is also distinct from neighbouring populations. This unique genetic profile challenges the routine assumption of large-scale population relocations by the Incas. Furthermore, speakers of Chachapoyas Quechua are found to share no particular genetic similarity or gene-flow with Quechua speakers elsewhere, suggesting that here the language spread primarily by cultural diffusion, not migration. Our results demonstrate how population genetics, when fully guided by the archaeological, historical and linguistic records, can inform multiple disciplines within anthropology.

## Introduction

Genetic studies have begun to contribute significantly to our understanding of the pre-colonial history of the Americas, and are able to fill in some of the gaps in the archaeological and historical records. Archaeology faces preservation biases between the diverse environments of the desert along the Pacific coast, the Andean highlands and the Amazonian rainforest. Written history in the Andes begins only with the Spanish conquest in the 1530s, and these first chronicles are fragmentary and contradictory.

Genetic results have returned important insights particularly on the macro-scale: the initial colonization events^1–3^ and the broad patterns of diversity, such as the genetic contrast between the ecological domains of the Andes and Amazonia^4,5^. Studies focused on sub-regions, however, face significant limitations when attempting to evaluate past population movements and contacts on a much finer scale, often due to the low resolution of the genetic markers selected, or to poor sample coverage. This study therefore explicitly selects high-resolution markers, and to improve sampling follows a strategy fully informed by historical, archaeological and linguistic contexts.

Our focus is on northern Peru, selected as a case-study for its significance in both environmental and historical terms. The Andes here are at their lowest elevation, and thus serve as a preferential corridor between Amazonia and the Pacific^6^. Towards the eastern slopes, where the environmental transition to Amazonia begins, is the cloud forest region of Chachapoyas^7^. The archaeological record here attests to a rich diversity of regional cultures over time, up until Inca conquest in the 1470s. ‘Chachapoya culture’ serves as a collective term for a series of political entities, independent but sharing common architecture, art (ceramic style) and iconography^8,9^. In the earliest historical accounts, Spanish chronicles make extensive mention of Chachapoyas for its long resistance to Inca conquest — and then as a clear example of the Inca state policy of forced resettlements. Chachapoyas is taken as one of few regions where the recalcitrant local population was essentially completely removed and replaced^10^ — making this a test-case for the ability of population genetics to challenge or confirm the (proto-)historical record, of questionable veracity here^10^ (91–118).

**Figure 1:**
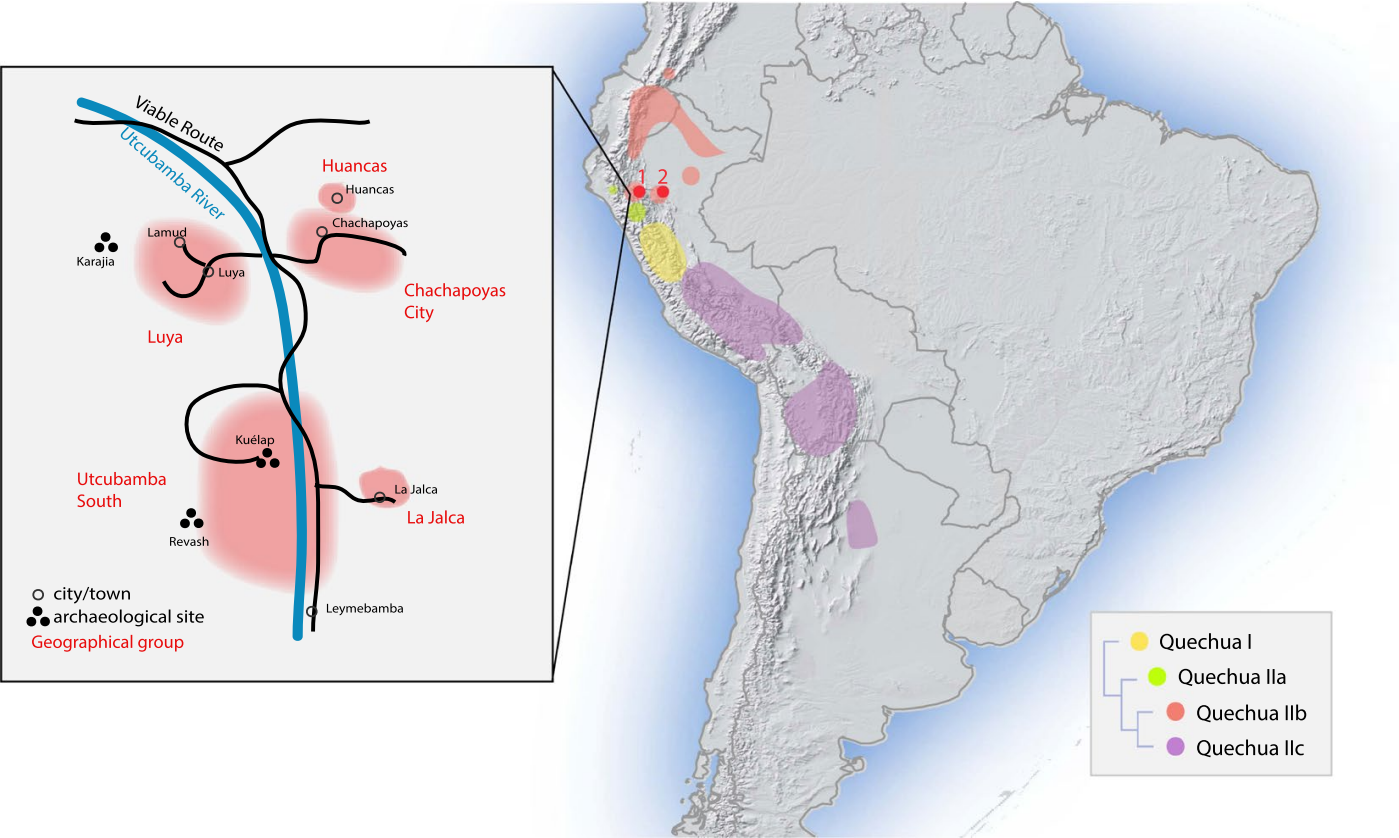
Map of sampling locations and approximate distribution of sub-branches of the Quechua language family, as traditionally classified (Adapted from^13^). Red dot 1 marks the sampling locations in the Amazonas region (Chachapoyas City, Luya, Huancas, Utcubamba South, La Jalca); red dot 2 marks that in the San Martín region (Lamas, Wayku neighbourhood). The inset zooms in on the sampling locations in Amazonas. Map generated in R - version 3.3.0 www.R-project.org/ ^62^. Raster physical map adapted from www.naturalearthdata.com/ *(*public domain).

The presumed native language of Chachapoya culture (referred to as “Chacha”) is extinct, although a few of its characteristics can be inferred from surviving placenames and surnames^11,12^. Quechua, meanwhile, never seems to have been dominantly established across northern Peru, and is spoken, in diverse local forms, only in sporadic pockets scattered across the region (Fig. 1). One of these pockets is Chachapoyas, where a small proportion of the present-day population, in a handful of small communities, still speaks a now moribund variety of Quechua that is difficult to classify within the family’s phylogeny. Another pocket of Quechua, also covered in this study, is the town of Lamas in San Martín province, in the Andean foothills at the edge of Amazonia. Both these forms of Quechua have usually been assigned to a putative ‘Quechua IIb’ branch, together with others spoken elsewhere in the Amazonian lowlands of northern Peru (in Loreto province), the ‘Inga’ spoken in southern Colombia, and the widely spoken Ecuadoran ‘Kichwa’^13^. Quechua did once serve as *lingua franca* for the Inca Empire, but that can explain only some of its diversity and distribution through the Andes. Other potential drivers include the forced population movements under the Incas, and the cultural and/or demographic impacts of earlier complex societies in the Andes, and of the Spanish colonial regime^14,15^. For further archaeological, historical and linguistic contextualization, see the Supplementary Text.

To introduce a genetic perspective on these scenarios from archaeology, history and linguistics, this study focuses on uniparental markers: i.e. mtDNA and the non-recombinant portion of the Y chromosome.

For mitochondrial DNA, in the Americas data are relatively abundant for the hypervariable region only. At a broad level, it is possible to identify variation in the frequencies of the four haplogroups most heavily represented in Native Americans (A2, B2, C1 and D1: for an overview see^16^). Beyond that, however, sequencing the hypervariable region alone is often insufficient to disentangle population relationships. This is a result of the Americas being settled only relatively recently, and through a bottleneck in Beringia^1,2^. Sequencing full mtDNA genomes gives an important gain in resolution, as demonstrated by recent studies that generate native mitogenomes for some parts of the Americas^17–21^. So by sequencing new sets of full mtDNA genomes, from regions of the continent selected for their distinctive local population prehistories, this study adds to our database for the Americas, and uncovers new patterns of genetic variation that bear directly on population prehistory here.

For the Y chromosome, standardized Short Tandem Repeat (STR) sets have been successfully applied to explore genetic diversity in the paternal line in South America^22–25^. The most widely used STR sets cover 12 or 17 markers, but newly available sets for 23 markers give more fine-grained results. Data from the Andean region reveal a nucleus of homogeneity across the central highlands, from central Peru to northern Bolivia, in populations speaking languages of the Quechua or Aymara families, which both likely originated within this broad region^13^. This pattern is confirmed in the mtDNA data^23,26^, and has typically been associated with the demographic impacts of the expansion and resettlement policies of the Inca Empire, and perhaps also of their most significant predecessor polities, Wari and Tiwanaku in the Middle Horizon period (c. 500–1050 AD)^24^.

Beyond this nucleus of homogeneity in the Andes, occasional populations in the Amazonian lowlands also speak the ‘highland’ language Quechua. Genetically, however, these populations do not show any particular relationship to the central highlands that would parallel this linguistic link. Quechua speakers from Lamas likely acquired the language by processes of cultural diffusion, not by significant migration from the highlands^25^. Finally, a recent study of 23 Y-chromosome STRs and the mtDNA HVSI has reported high genetic diversity in populations from Chachapoyas and surrounding areas^27^. It remains to be clarified, however, what the relationships are between Chachapoyas and other parts of the Andes, which we test here using more and higher-resolution genetic data from Chachapoyas.

For this study, we generated 23-STR profiles and full mtDNA genomes from Quechua-speaking populations of northern Peru, with the aim of exploring the impacts of the Inca period, the possible genetic inheritance from pre-Inca periods, and the dynamics behind the diffusion and differentiation of the multiple Quechua varieties scattered across the region. We sampled 119 individuals, with an explicit strategy targeting firstly the surviving Quechua-speaking pockets in Chachapoyas and Lamas. Secondly, we focused on surnames characteristic of putative “Chacha” linguistic origin (i.e. neither Spanish nor Quechua), to try to trace back to a possible genetic legacy of the Chachapoya population from before the Inca conquest (Supplementary Text and Supplementary Table S1).

We uncover an enclave of diversity on a micro-geographical scale in both Y-chromosome and mtDNA, independent of the main network of intense genetic (and linguistic) interaction in the south-central Andes. Our findings have multiple implications for Andean (pre)history. Firstly, we find evidence to challenge the presumption of major Inca impacts and resettlements affecting the Chachapoyas population. Secondly, we find no direct demographic connections between populations that speak varieties of the disputed QIIb clade of Quechua, and support a cultural rather than demographic model for its spread in northern Peru.

## Results

### mtDNA

**Figure 2:**
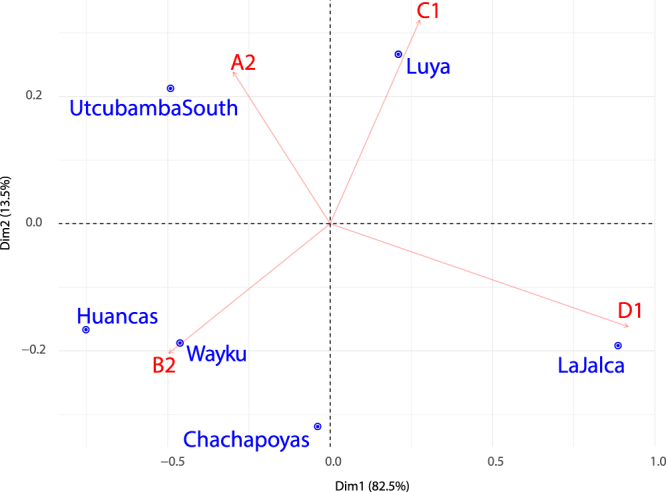
CA plot of mtDNA haplogroup frequencies.

Full mitochondrial genomes were successfully sequenced for 116 individuals (data deposited in GenBank under accession numbers MG571104 - MG571221). Of these, 113 could be assigned (given the local origins of their parents and grandparents) to one of six broadly-defined geographical groups (Fig. 1, Supplementary Table S1). Sample sizes range from eight individuals in the Chachapoyas city group to 36 in the Luya group. All except two individuals have Native American haplogroups: most frequent in our sample is B2 (35%), followed by A2 (26%), D (D1 + D4h3 23%) and C1 (16%). Differences in haplogroup compositions are summarized in a CA plot (Fig. 2). Haplogroup frequencies were also compared to those of populations from across South America in a broader CA plot (Supplementary Fig. S1). Each group is more similar in haplogroup composition to other groups outside the region than to any other group within Chachapoyas itself.

Full mtDNA genomes were first screened for diversity within each group, comparing nucleotide diversity and haplotype diversity (Supplementary Table S2). For both values of diversity, the same trend emerges: Luya, Chachapoyas and Utcubamba South are the most diverse, Huancas the least diverse. Between populations, ϕ_ST_ genetic distances (Supplementary Table S2) indicate that La Jalca has a highly distinctive genetic make-up, showing the highest distance to other groups, in particular to Huancas, Wayku and Utcubamba South.

We constructed Networks (Supplementary Fig. S2, Supplementary Text) to compare the diversity of our sample with other full mtDNA sequences available from the literature. Our samples either stand alone, or group in branches mostly with each other. Only rarely do they fall together with samples drawn from the literature, in a shared branch. This effect can be in principle explained by our target populations having remained relatively isolated from other sampling localities in our database, although it may also reflect lack of adequate sampling in the literature, and/or extinction of more closely related populations in the post-colonial era^20^.

**Figure 3:**
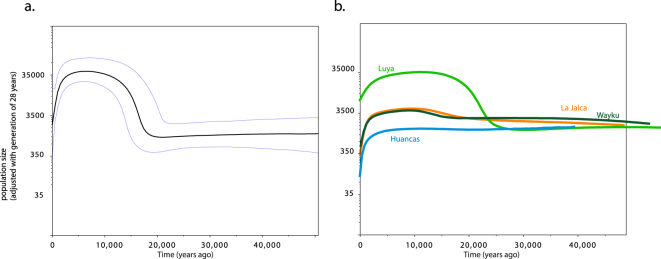
(**a**) BSP for mtDNA sequences for the whole sample. (**b**) BSP for mtDNA sequences for populations with sample size > 14.

BEAST was used to construct a tree genealogy and Bayesian Skyline Plots (BSPs). The BSP for all the sequences generated in this study displays a steep curve indicative of an expansion starting ~17,000 years ago (kya): the High Probability Density (HPD) ranges from 15 to 22 kya (Fig. 3a), in line with mainstream views on the timing of the first settlement of the Americas^1,20^. The increase in population size across our whole sample is more than 33-fold: for a generation time of 28 years^28^, this corresponds to an increase in effective population size (N_e_) from 1000 to 35,000 individuals. BSPs for the individual population groups (Fig. 3b) do reveal some differences: La Jalca and Wayku show a fairly stable population size, with but a faint indication of increase through time. Luya instead clearly reproduces the increase in population size observed for the whole dataset.

**Figure 4:**
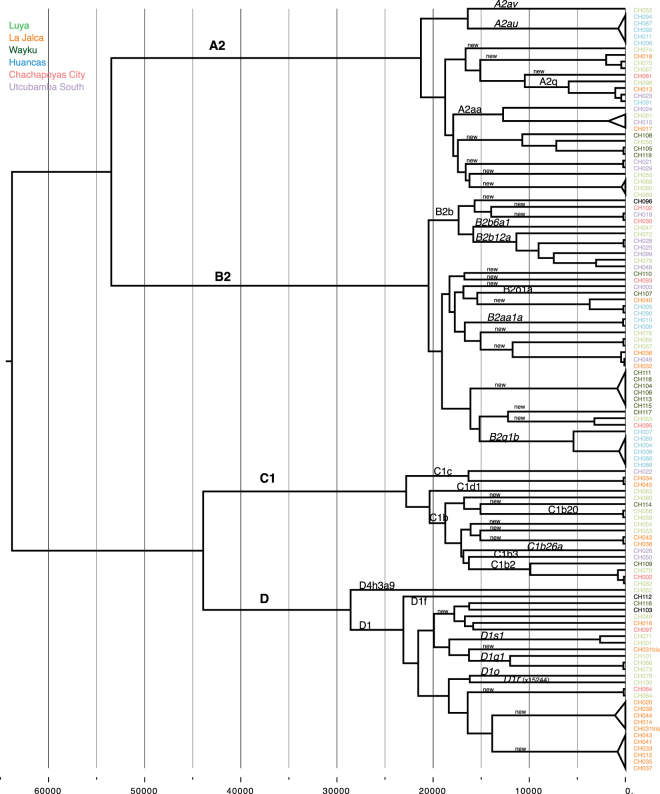
Annotated tree of mtDNA sequences generated with BEAST. Branches are named after Phylotree v.17^29^; branches in italics correspond to the nomenclature recently proposed by^19^. Individual samples are colour-coded according to the sub-population affiliation.

The annotated tree from the BEAST runs (Fig. 4) gives age estimates for the sequence of major branching events, and mirrors the signal of expansion in the BSP, and its time-depth, with a strong effect of branch divergence between 15 and 20 kya. Some cases of more recent divergence are found at the tips of the tree, in closely related sequences, clustered together in a triangle span for illustrative purposes. The divergence time for these sequences is estimated at roughly within the last 1000 years. One notable aspect of the results, even if not unexpected when sequencing mitogenomes from an understudied region of the Americas, is that a large proportion of the branches we report here represent new sub-haplogroups not yet identified in previous research (verified against Phylotree build 17^29^ and a recent review^19^). Within A2, B2 and D1 we find 27 previously unreported branches with divergence times ≥15,000 years ago, i.e. possibly shortly after first human entry to the Americas. Only within C1 could all the lineages be assigned to already known sub-haplogroups: C1b, C1c and C1d.

### Y chromosome

Male individuals were first genotyped for haplogroup assignment; only those belonging to haplogroup Q, the characteristic Native American marker of interest for our study, were then typed for STR haplotypes and included in the intra- and inter-population analyses. The other Native American haplogroup, C2-M217, was not found in our sample. The remaining samples belong to broad macro-haplogroups other than Q, as detailed in the Supplementary Text. Haplogroup frequencies for each population are included in Table S2. The highest frequency of non-Native haplogroups is in La Jalca (40%), the lowest in Luya (20%).

Between population comparisons at a regional scale are based on the 23-loci dataset, which includes the sample from Guevara *et al*.^27^. The haplotype sharing plot (Supplementary Fig. S3) and the Median Joining network (Supplementary Fig. S4) show that sharing is limited (139 unique haplotypes over 175 individuals, of which only 13 are shared between two or more populations) and that several branches are highly localized (Supplementary Text). These local branches correspond in some cases to samples marked for non-Spanish surnames (of “Chacha” or Quechua origin – Supplementary Fig. S5).

After controlling for non-significant R_ST_ values, amount of haplotype sharing and compatible diversity values, we merged some of our smallest samples with other related ones, to allow for continent-wide comparisons. The Chachapoya sample from Guevara *et al*. was merged with our small Chachapoyas city group, the Huancas sample from Guevara *et al*. with our Huancas, and finally the Quechua Lamas sample from Sandoval *et al*.^25^ (only 17 loci) with our Wayku.

Between population comparisons at a continental scale are based on 17 or 15 loci. Supplementary Table S3 lists the 90 populations included and their diversity values. The least diverse of our samples (partially merged with Guevara *et al*. and Sandoval *et al*.) are those from Huancas, La Jalca and Wayku: their low variance is similar to that found in populations predominantly from Amazonia. Higher values for within population variance (but close to the average for the continent) are found for the Chachapoyas city, Luya and Utcubamba South groups. Haplotype diversity values follow the same trend.

**Figure 5:**
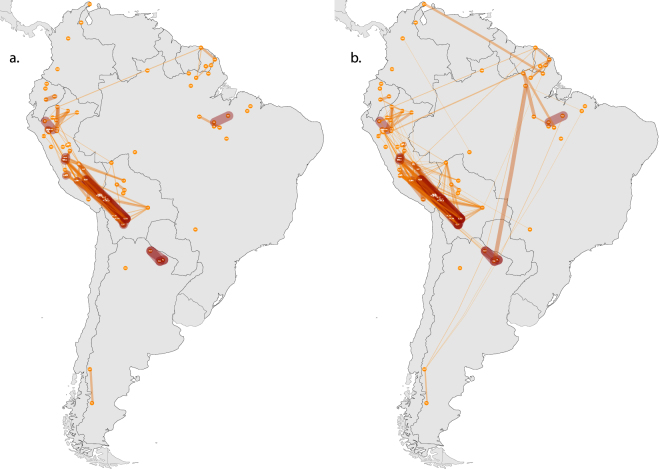
Map depicting patterns of Y chromosome haplotype sharing at a continental scale. Thin yellow lines indicate the lowest levels of exchange (from just a single pair of individuals sharing an identical or similar haplotype); thick red lines the highest (up to a maximum of 154 identical and 310 similar haplotypes shared between the Toba and Pilaga samples). (**a**) Sharing of identical haplotypes. (**b**) Sharing of similar haplotypes (allowing 1 step mutation in loci with a high mutation rate – see Methods). Map generated in R - version 3.3.0 (www.R-project.org/)^62^.

To identify close connections between populations, identical haplotypes were annotated with two methods: first by a heatplot of sharing between relevant populations from Ecuador and Peru, based on 17 loci (Supplementary Fig. S6), then in a map to display the full amount of sharing throughout the continent, based on 15 stable loci, weighted for their mutation rates (Fig. 5). The patterns of sharing do not support any recent connection to speakers of other ‘Quechua IIb’ varieties. The Wayku do show affinities to some other Quechua speakers: those in north-east Peruvian Amazonia (see Supplementary Text for details).

The continental sharing map helps visualize connections between the Chachapoyas region and its close and distant neighbours. The two maps in Fig. 5 show shared identical haplotypes (Fig. 5a), and shared similar haplotypes, i.e. allowing for one stepwise mutation in the more rapidly mutating loci (Fig. 5b). Supplementary Fig. S7 zooms in on the region of interest, and shows a limited amount of sharing involving the target populations near Chachapoyas (in blue, four cases of direct sharing which involve Utcubamba South). This stands in stark contrast to the nucleus of homogeneity in the highlands, across the southern half of Peru and Bolivia, where rates of identical and similar haplotypes are remarkably high, creating a dense network of exchange (see Supplementary Text for details on haplotype sharing).

Supplementary Fig. S8 visualizes the correlation between the frequency of shared haplotypes and the sample size of each population. The sample from Chachapoyas City shares relatively few haplotypes for its relatively large sample size, and is more isolated than average Amazonian populations when sharing between close neighbours is ruled out (Supplementary Fig. S8b). Sharing in Chachapoyas is lower than in the Andes but higher than in Amazonia when sharing with close neighbours is not excluded (Supplementary Fig. S8a). The sharing pattern is then decomposed to the regional level, by dividing the South American samples into 10 groups (Supplementary Fig. S9a) and focusing on five linguistic groups of interest for the Andean region (Supplementary Fig. S9b). The highest level of sharing involves the Central South Andes, and the speakers of Quechua IIc: the two groups include almost the same samples (Supplementary Fig. S9c,d). Finally, coalescent simulations were applied to estimate the incidence of haplotype sharing between two populations with varying N_e_, time of divergence, and symmetric migration rate. The results reveal that the target case of “no haplotype sharing”, as observed between Chachapoyas city and the other published samples, is detectable (>5% of cases) until 10 generations split time and N_e_ > 1000. For more recent split times and N_e_ ≤ 1000, instead, haplotype sharing would be predominant (>95% of cases; Fig. S10). When migration between the two populations is considered, “no haplotype sharing” could still be detected with appreciable frequency only for large N_e_ (3000)(Fig. S10d), while for smaller N_e_ “no haplotype sharing” is detectable for a maximum migration rate of 0.015 (N_e_ = 1000, Fig. S10c) or 0.005 (N_e_ = 500, Fig. S10b).

## Discussion

First we look at a continental scale, and the position of the population of Chachapoyas and Lamas (Wayku) within the Americas. On this broadest level, our high-resolution analysis of uniparental markers reveals a pronounced (high frequency of Native American haplogroups) and distinctive (unique to the region, in particular in Chachapoyas) native component. Our dataset of 114 Native American mtDNA genomes corresponds to a new hotspot of diversity, and suggests that other regions of South America may likewise harbour pockets of diversity that have so far remained undetected.

Recent publications of mtDNA genomes have mostly focused on specific lineages of interest, misrepresenting the diversity at the population level at the fullest resolution for this (maternally inherited) marker. Our new dataset has not only been able to distinguish characteristic lineages for the population groups involved, but has also uncovered notable diversity within the four major Native American mtDNA haplogroups. In the BEAST tree reconstruction (Fig. 4), 65 of these new sequences within A2, B2 and D are sub-haplogroups newly identified here, i.e. they cannot be assigned to any of those already defined in previous studies (Phylotree v. 17^29^ and the latest publication by Brandini *et al.* ^19^).

This result entails two significant findings: a) Previous studies have underestimated the extent of diversity and geographical differentiation within Native American mtDNA lineages; and b) Despite the generally low diversity caused by the strong founding bottleneck in Beringia, the significantly higher resolution available from full mitogenome analysis over the HVSI does make it possible to reveal demographic dynamics at a regional scale. As illustrated in Fig. 4, each of the six groups contains exclusive sub-branches in the network, a result compatible with residence patterns localised here over time.

Secondly, we compared our results against competing hypotheses on the origins of the Chachapoyas population: an allochthonous origin (either from the lowlands, the highlands or from several localities, as a result of the Inca resettlement policies) or an autochtonous one (see Supplementary Text). To explore these hypotheses, the present-day genetic data can be interrogated for any signals to support connections to other regions, and to assess whether the genetic make-up of Chachapoyas has or has not been relatively stable through time. Since mitogenome data from the Andes are still too scarce, we considered the Y-chromosome STR dataset (of 90 populations), with the caveat that it reflects only paternal population history. In principle, the high internal diversity of our sample could be compatible with populations relocated here from multiple, distinct and far-flung regions of the Inca empire or indeed from earlier highland polities. Our sample from the Chachapoyas region turns out not to be genetically close, however, to the core Andean exchange network in the highlands of southern Peru and Bolivia, sharing only four haplotypes with Utcubamba South (Fig. 5, Supplementary Figs S6,S7,S8,S9c). This result is unexpected for what is considered a strategic crossroads region, a corridor from Amazonia to the Andes^6^, and particularly in comparison to the conspicuous amount of sharing found in other populations, for example the Yanesha of central Peru, supposedly more isolated both geographically and culturally^24^. The high-altitude Yanesha, with a sample size of 55 individuals, share haplotypes with 20 other populations (the highest number in our continental dataset). While occasional other populations in the database display a unique genetic profile with no haplotype sharing (Fig. S8), the general sharing trend is higher in all neighbouring regions (Fig. S9).

A recent allochthonous origin would entail some level of haplotype exchange, and our sample size is large enough that any such exchange should be detected — but we do not find this (Supplementary Fig. S8). It is highly improbable that no shared haplotypes would be reported for a population split within the past 20 generations (~600 years), as our simulations confirm for a compatibly small N_e_ of 500 or 1000 (Fig. S10). This time constraint argues against the scenario of a complete relocation forced by the Incas or by subsequent Spanish colonial policies. The hypothesis most directly and parsimoniously compatible with the distinctiveness of our Chachapoyas sample seems to be that of autochthonous development and continuity over the generations (see Supplementary Text for further discussion). Nevertheless, more ancient connections to the lowlands and/or the highlands cannot be ruled out, given the fast mutation rate of the Y chromosome and the overall low diversity of the Native American genetic component. The ultimate tool to test and refine these interpretations would be the analysis of ancient DNA from both before and after Inca conquest.

The support for local continuity over at least the last 20 generations (Fig. S10) allows us to link the current genetic makeup of the region back to the immediately pre-Inca period, and explore microgeographical diversity patterns. Our six sub-regions display a considerable degree of population structure for such a small sample area: this is reflected by the presence of characteristic lineages for both Y chromosome and mtDNA (see networks in Figs S2 and S4). In mtDNA the samples from Luya and La Jalca (which have the largest population sizes) are distinct in haplogroup frequencies (Fig. 2, Supplementary Fig. S1), in internal diversity (Supplementary Table S2), in projected population sizes (Fig. 3b) and in admixture histories (see Supplementary text). This fine population structure within the provinces of Chachapoyas and Luya appears consistent with the archaeological record, which points to a collection of societies forming a shared ‘Chachapoyas culture’, but without a single over-arching political unit^8^. Obviously, drift has exacerbated differences in haplogroup frequencies, especially through the bottleneck after European contact, when widespread epidemics led to a dramatic loss of native lineages throughout the Americas^20^.

Thirdly, our data can also test between hypotheses on the dispersal and phylogeny of Quechua. In sampling, we targeted localities in Chachapoyas identified as recently or still currently Quechua-speaking (Supplementary Table 1, Supplementary Text). Our results can thus provide a new perspective on which demographic processes may connect the various Quechua-speaking populations across northern Peru and beyond, particularly those traditionally classed within Quechua IIb. We focused on haplotype-sharing patterns, to restrict the time-depth of our analysis to population connections within historical times here (see above), adjusting for the mutation rates of the markers used (see Methods).

A primary finding from our linguistically-targeted sampling is that the samples from Chachapoyas and Lamas are not genetically connected, and that no major connections appear between speakers of the different varieties of the putative Quechua IIb branch overall (Supplementary Figs 6 and 7). Despite our large sample size from Chachapoyas and adjacent provinces, the level of haplotype sharing with other populations is nil, even with their supposedly close linguistic relatives — the only exception is Utcubamba South, which does share haplotypes with three populations outside the province. Rather, the speakers from Wayku share a network of connections with other lowland populations (Supplementary Figs S7,S9c). Nor does our Chachapoyas sample share haplotypes with Quechua-speakers in the Ecuadorian lowlands (‘QIIb’) — who instead share haplotypes with other lowland populations, and have a typically Amazonian genetic profile^24,25^ (Fig. S9c,d).

This rules out any strong demographic connection between speakers of the disparate varieties of Quechua all traditionally classified into the IIb clade, and bears on the open question of when and how Quechua came to be spoken in northern Peru, Ecuador and southern Colombia. In the case of Wayku, historical sources report how Jesuit and Franciscan missionaries from Quito purposely introduced Quechua as a *lingua franca* in the *Reducciones* (mission stations) that they established among various tribes of the Marañón, Napo and Amazon rivers^30,31^. This finding is compatible with recent genetic analyses of the Wayku population as predominantly local Amazonian^25^. For Chachapoyas, however, there is little parallel documentary evidence for a missionary diffusion model, and the alternative explanation remains that Quechua arrived here with at least some population resettled here by the Incas, from some other part(s) of their Empire. But in either scenario, the genetic data offer no support for the existence of any putative single Proto-QIIb language, whose speakers could have been a common migration source for the populations that today speak the Quechua varieties of Ecuador, Colombia, Chachapoyas and Wayku.

Zooming out to the rest of northern Peru, there are only two other Quechua-speaking groups in the region, both traditionally classified within a hotly disputed ‘QIIa’ clade. Only for one of these — Cajamarca — does the genetic literature include samples that may represent speakers of the local Quechua (or their descendants). Again, however, our Chachapoyas sample shows no connections to the Cajamarca sample, which itself shows no connections to other Quechua-speakers in northern Peru (Fig. S9d), but only to the (linguistically unrelated) Jívaro from Amazonia.

Finally, zooming out further still, the origin of the Quechua language lineage as a whole is generally set in the south-central highlands of Peru^13^. That linguistic homeland falls within the network of very high homogeneity in the gene pool and high levels of haplotype sharing across the highlands of southern Peru and Bolivia, as already reported in the literature^23,24,26,32^ (Fig. S9d). This is the core territory of the best known form of Quechua, with by far the largest number of speakers: ‘Southern Quechua’, traditionally classified as a QIIc clade. The genetic network also includes all speakers of Aymara in this region, and is consistent, furthermore, with the intense contact, shift and convergence between the distinct Quechua and Aymara language lineages^33^.

Although the uniparental markers are analysed here to high resolution, even so they still constitute only a limited perspective on population history, which thus may underrate the amount of contact in the region of interest. It is conceivable that high-throughput genomic data may reveal more connections that bear on both the origin of Chachapoyas populations and any relationships among Quechua-speaking groups across northern Peru. This would entail extensive and targeted sampling coverage, however, from both these regions and the broader Andean context.

In conclusion, with a targeted sampling strategy focused on tracing autochthonous surnames and local Quechua-speaking survivals, we have been able to uncover new genetic variation, and a potential signature of continuity through multiple layers of history. High-resolution uniparental markers prove able to disentangle relationships not just over the Andean region as a whole, but also at a local scale, despite the relatively low overall genetic diversity so often regarded as an obstacle to recovering fine-grained population history in the Americas. The long and complex (pre)history of Andean civilization has left traces that can be recovered not only from a rich archaeological record and a complex linguistic panorama, but also from the details of the genetic makeup of Andean populations living there today. This precision also helps clarify models of the diffusion of the largest surviving language family of the entire Americas, Quechua. Clearly, those models need to combine two contrasting mechanisms: significant demographic exchange in the south, and language shift by predominantly cultural forces in the north. To further test this double scenario, and to gain a full insight into the genetic ancestry of the region, this study should ideally be complemented by autosomal genomic data. Finally, the full mtDNA genome dataset released here will stand as a valuable resource for broader, continent-wide comparisons, and for ancient DNA studies — for which the case-study region of Chachapoyas holds out particular potential.

## Methods

### Sample design and strategy: genealogical and linguistic characterization

This work is the result of a fieldwork expedition conducted in 2015 to the regions of Amazonas and San Martín, with the support of local political authorities and cultural representatives. Our sampling strategy was guided by linguistic indicators that parallel, and can help tease apart, the three main layers of history in the region: the Chachapoya autochthonous substrate; the brief period of Inca influence and then dominance, from the 1470s to the 1530s, which presumably fostered the use of Quechua; and finally Spanish rule immediately thereafter.

Linguistic research directed our sampling in two ways: firstly it enabled us to focus on the Native American genetic component, and secondly, within the native component, it allowed us to further distinguish between Chacha and Quechua, most plausibly corresponding to periods before and after Inca impacts here. The presence of Quechua, surviving in just a few villages scattered across Chachapoyas, enabled us to target sampling on those locations. In each one, we conducted a survey to assess how far Quechua was (or still is) spoken in each participant’s family, and to identify the birthplaces of parents and grandparents. We also conducted extensive archival work on historical patterns in local surname origins, following not just Quechua markers but trace indicators of the now extinct Chacha language, too. Supplementary Table S1 shows our results by presence of Quechua and by surname origin, with further details on linguistic and surname characterization given in the Supplementary Text.

### The genetic sample

Saliva samples were collected from healthy volunteers. Each participant signed a written consent form, after being fully informed of the purpose of the study, with the opportunity to ask questions for further clarification. The project and the informed consent were approved by the Ethics Committee of the University of San Martín de Porres, Lima (Comité Institucional de Ética en Investigación de la Universidad de San Martín de Porres — Clínica Cada Mujer, Ofício No. 579-2015-CIEI-USMP-CCM, 12/05/2015) and by the Ethics Committee of the University of Jena (Ethik-Kommission des Universitätsklinikums Jena, Bearbeitungs Nr. 4840-06/16). All methods were performed in accordance with the relevant guidelines and regulations. The sample analysed in this study represents only a small fraction of the population living in the provinces of Chachapoyas and Luya, and in Wayku, and so is only partially representative of the complex demographic history of these regions and of their inhabitants’ ancestors.

For Y-chromosome and mitochondrial analysis, individuals were assigned to different regional groups according to their self-identified paternal or maternal ancestry (up to two generations): Huancas, La Jalca, Chachapoyas city, Utcubamba South, Luya. These locations are all in the Amazonas region; our final sample is from the region of San Martín, specifically from the Wayku neighbourhood in Lamas. (For further details on sample locations, see the map in Fig. 1 and the Supplementary Text). Information on the 119 individuals sampled is given in Supplementary Table 1. The precise identification of their home villages is not given, however, in order to guarantee anonymity. Individuals marked “OUT” have maternal or paternal ancestry from other regions of Peru, outside the provinces that are the focus of this study, and were excluded from the group comparisons.

DNA was extracted with QIAamp DNA Mini Kits (Qiagen) according to the manufacturer’s protocol.

### mtDNA

Libraries were prepared with a multiplex protocol for the Illumina Genome Analyzer platform, and enriched for mtDNA with in-solution capture following standard protocols as performed in previous studies^34,35^. Libraries were pooled and sequenced in Illumina HiSeq. 2500 on two Rapid lanes with 100 + 7 + 100 + 7 cycles. The Kit for the chemistry was V2.

Base-calling was performed using freeIbis^36^, and reads were processed as follows^37,38^: read adaptors were trimmed, and reads were filtered for at most 5 bases with a quality score <15, and indexes for no bases with quality scores <10. Sequences were manually checked with Bioedit (www.mbio.nc-su.edu/BioEdit/bioedit.html). Average coverage was ~3000X, with a minimum of 58X and a maximum of 12,093X.

The two poly-C regions (np 303–315, 16183–16194), prone to sequencing errors, were trimmed from the final alignment used in the analysis. Sequence alignments, including the RSRS sequence^39^, were assembled with MAFFT v7.123b^40^.

Haplogroup assignment was performed with Haplofind^41^ and Haplogrep 2^42^, and manually confirmed by checking diagnostic positions as described in Phylotree v.17^29^. Haplogrep was also used to list the polymorphisms in each sample, as included in Table S1.

Two sets of comparative data were collected from the literature: a dataset of haplogroup frequencies from across the Americas, adapted from^24^ (see original publication for population labels), and a dataset of full genome mtDNA sequences^2,17–19,39,43–54^. The comparative sequence dataset includes a total of 725 sequences from haplogroups A, B, C and D, from North America, Mesoamerica and South America, and was screened to minimize the number of missing sites. The maximum number of missing sites allowed per sequence was 2. The list of sequences included, with references to the corresponding publications, is available in Table S4.

### Y chromosome

Eighty-eight male individuals were typed with a basal *SNaPshot* ^®^ multiplex, as described in^55^. The Q samples were also tested for markers downstream of M3: Y14998418(MG2), PV2, SA01, M19, M557^55,56^. Samples belonging to haplogroup Q were typed with the PowerPlex^®^ Y23 System (Promega, Mannheim, Germany) as previously described^57^. The data were analyzed with GeneMapper^®^ ID-X1.1.1. (Life Technologies, Darmstadt, Germany). Haplogroup nomenclature follows van Oven *et al*.^58^ where possible, with the name of the diagnostic marker following the conventional name of the haplogroup. Haplogroup affiliations and STR profiles are reported in Supplementary Table 1.

Intra-population comparisons were performed on a set of 23 or 17 loci. Comparisons with 23 loci were performed with the analogous dataset from^27^, which included other individuals from Chachapoyas (some from La Jalca and some from the province of Luya) and Huancas, as well as neighbouring populations from Cajamarca in the highlands, and the Jívaro in Amazonia. The dataset from Guevara *et al*. was filtered for individuals assigned to haplogroup Q with more than 90% accuracy from the haplogroup predictor. The resulting dataset for 23 loci included a total of 177 individuals. Continent-wide comparisons were performed with a subset of 17 loci, to include further data available from the literature, for a total of 90 populations^22–25,32,59–61^.

### Computational analysis

Values of diversity, ϕ_ST_, haplogroup and haplotype comparisons, haplotype sharing and correlations between features of the dataset were calculated and plotted in R^62^ using the packages Pegas^63^, factoextra^64^, MASS^65^, vegan^66^, ape^67^, maps^68^, geosphere^69^, ggplot2^70^ and ggmap^71^. Median-joining networks were calculated with Network 4.6.1.3 (Fluxus Technology, http://www.fluxus-engineering.com) and plotted with Network Publisher. In the Network analysis of the STR haplotypes, weights were assigned to each individual STR locus in inverse proportion to the variance observed in our dataset. Individuals from the published dataset who were missing values for one or more loci were excluded from this analysis. Direct haplotype sharing was performed with the 23 and 17 loci datasets and visualized in heatplot maps. The maps showing amounts of shared identical and similar haplotypes were calculated for a subset of 15 loci (excluding unstable loci DSY385a and b), adjusted for the mutation rate for each locus (mutation rates from https://yhrd.org/). The similar profiles allow up to one mutation in one of five loci DYS389II, DYS439, DYS456, DYS458 and DYS635 with mutation rates higher than 0.003 mutations per year per generation.

Phylogenetic trees and Bayesian Skyline Plots (BSPs) were generated with BEAST v1.8^72^. BEAST runs were performed with full mtDNA genomes, for the entire dataset as well as for individual populations. The dataset was partitioned into the coding region, to which we assigned a rate of 1.708 × 10^−8^ substitutions per nucleotide per year, and the non-coding region, to which we assigned a rate of 9.883 × 10^−8^ substitutions per nucleotide per year^73^. The best substitution model for each partition and for each population subset was determined using jModelTest v2.1.7^74^. In order to determine the best clock model and tree model, different runs were performed with BEAST and evaluated by a Bayes Factor (BF) analysis^75^. For the clock model, we compared a strict clock model, a relaxed exponential, an uncorrelated relaxed lognormal (ULN) clock model and a random clock.

The best substitution models determined by jModelTest for the whole dataset were TNI + Invariant Sites for the coding part of the alignment, and HKI + Invariant Sites + gamma for the non-coding part. Maximum likelihood estimates for the different combinations of clock models were evaluated by BF analysis^76^, and showed decisive support for a ULN clock for the coding region and a random clock for the D-Loop. The Bayesian Skyline tree model was chosen in order to display the BSP (given the strong expansion shown by the Native American sequences, a constant size model was not considered). A total of 50 million chains were executed for the entire sequence set, to ensure reliable ESS values. For the single populations set, we performed 10–20 million chains. Multiple runs were performed on each dataset, and combined using logCombiner. The maximum clade credibility was determined using TreeAnnotator and visualized with FigTree (http://tree.bio.ed.ac.uk/software/figtree/).

Coalescent simulations for 15 Y chromosome STRs were performed with Simcoal v.2.1.2 (http://cmpg.unibe.ch/software/simcoal2/), with the mutation rates used above, a stepwise mutation model with a geometric parameter of 0.1, and a simple population history of two populations (pop1 sample size 70, corresponding to Chachapoyas, and pop2 sample size 20, chosen as a rounding down of the average sample size of 23) coalescing at a given split time T1 with no growth rate. We performed a total of 800 runs of 100 simulations each with varying conditions: T1 between 10 and 200 generations, N_e_ for pop1 and pop2 between 500 and 5000, and symmetric mutation rate between pop1 and pop2 between 0 and 0.1. For each simulation, we recorded the presence of haplotype sharing events, to give a percentage of sharing events for each simulated condition.

### Data availability

The STR and mtDNA haplotypes generated during this study are included in the Supplementary Information files. The whole mtDNA sequences are available in GenBank under accession numbers MG571104 - MG571221.

## Electronic supplementary material

Supplementary Material

Supplementary Tables

## References

[1] Kitchen, A., Miyamoto, M. M. & Mulligan, C. J. A three-stage colonization model for the peopling of the Americas. *PLoS One* **3** (2008). 10.1371/journal.pone.0001596 PMC2223069 18270583

[2] Tamm, E. *et al*. Beringian standstill and spread of Native American founders. *PLoS One* **2** (2007). 10.1371/journal.pone.0000829 PMC1952074 17786201

[3] Raghavan, M. *et al*. Genomic evidence for the Pleistocene and recent population history of Native Americans. *Science* (*80-*.). 1–20, 10.1126/science.aab3884 (2015). 10.1126/science.aab3884 PMC4733658 26198033

[4] Tarazona-Santos E (2001). Genetic Differentiation in South Amerindians Is Related to Environmental and Cultural Diversity: Evidence from the Y Chromosome. Am. J. Hum. Genet..

[5] Wang, S. *et al*. Genetic Variation and Population Structure in Native Americans. *PLoS Genet* **3** (2007). 10.1371/journal.pgen.0030185 PMC2082466 18039031

[6] Lathrap DW (1973). The antiquity and importance of long-distance trade relationships in the moist tropics of pre-Columbian South America. World Archaeol..

[7] Muscutt, K. *Warriors of the Clouds: A Lost Civilization in the Upper Amazon of Peru*. *Univ. New Mex. Press, Albuquerque* (University of New Mexico Press, 1998).

[8] Church, W. & Von Hagen, A. In *The Handbook of South American Archaeology* (eds. Silverman, H. & Isbell, W. H.) 903–926 (Springer, 2008).

[9] Bandelier, A. *The Indians and aboriginal ruins near Chachapoyas in northern Peru*. (Historical Records and Studies, 1907).

[10] D’Altroy, T. N. *The Incas*. (John Wiley & Sons, 2014).

[11] Taylor, G. *Estudios lingüísticos sobre Chachapoyas*. *Travaux de l’IFEA* (2000).

[12] Valqui Culqui, J. Reconstrucción de la lengua chacha mediante un estudio toponímico en el distrito de La Jalca Grande (Chachapoyas-Amazonas). (University of Lima, 2004).

[13] Cerrón-Palomino, R. *Lingüística Quechua. 2nd ed*. (Bartolomé de Las Casas, 2003).

[14] Adelaar, W. F. H. & Muysken, P. C. *The Languages of the Andes*. (Cambridge University Press, 2004).

[15] Heggarty P (2007). Linguistics for Archaeologists: Principles, Methods and the Case of the Incas. Cambridge Archaeol. J..

[16] Bisso-Machado R, Bortolini MC, Salzano FM (2012). Uniparental genetic markers in South Amerindians. Genet. Mol. Biol..

[17] de Saint Pierre M (2012). An alternative model for the early peopling of Southern South America revealed by analyses of three mitochondrial DNA haplogroups. PLoS One.

[18] Cardoso S (2012). Genetic uniqueness of the Waorani tribe from the Ecuadorian Amazon. Heredity (Edinb)..

[19] Brandini, S. *et al*. The Paleo-Indian Entry into South America According to Mitogenomes. *Mol. Biol. Evol*. 10.1093/molbev/msx267 (2017). 10.1093/molbev/msx267 PMC5850732 29099937

[20] Llamas, B. *et al*. Ancient mitochondrial DNA provides high-resolution time scale of the peopling of the Americas. *Sci. Adv*. **2** (2016). 10.1126/sciadv.1501385 PMC4820370 27051878

[21] Arias, L., Barbieri, C., Barreto, G., Stoneking, M. & Pakendorf, B. High-resolution mitochondrial DNA analysis sheds light on human diversity, cultural interactions, and population mobility in Northwestern Amazonia. *Am. J. Phys. Anthropol*. 10.1002/ajpa.23345 (2017). 10.1002/ajpa.23345 29076529

[22] Roewer, L. *et al*. Continent-wide decoupling of Y-chromosomal genetic variation from language and geography in native South Americans. *PLoS Genet*. **9** (2013). 10.1371/journal.pgen.1003460 PMC3623769 23593040

[23] Sandoval JR (2013). The genetic history of indigenous populations of the Peruvian and Bolivian Altiplano: the legacy of the Uros. PLoS One.

[24] Barbieri C (2014). Between Andes and Amazon: The genetic profile of the Arawak-speaking Yanesha. Am. J. Phys. Anthropol..

[25] Sandoval JR (2016). The Genetic History of Peruvian Quechua-Lamistas and Chankas: Uniparental DNA Patterns among Autochthonous Amazonian and Andean Populations. Ann. Hum. Genet..

[26] Barbieri C, Heggarty P, Castrì L, Luiselli D, Pettener D (2011). Mitochondrial DNA variability in the Titicaca basin: Matches and mismatches with linguistics and ethnohistory. Am. J. Hum. Biol..

[27] Guevara EK, Palo JU, Guillén S, Sajantila A (2016). MtDNA and Y-chromosomal diversity in the Chachapoya, a population from the northeast Peruvian Andes-Amazon divide. Am. J. Hum. Biol..

[28] Fenner JN (2005). Cross-cultural estimation of the human generation interval for use in genetics-based population divergence studies. Am. J. Phys. Anthropol..

[29] van Oven M (2015). PhyloTree Build 17: Growing the human mitochondrial DNA tree. Forensic Sci. Int. Genet. Suppl. Ser..

[30] García, L. *Historia de las misiones en la Amazonia Ecuatorian*a (1999).

[31] San Román, J. *Perfiles históricos de la Amazonía peruana, 2da. ed*. (CETA, CAAAP, IIAP, 1994).

[32] Gayà-Vidal M (2011). MtDNA and Y-chromosome diversity in Aymaras and Quechuas from Bolivia: Different stories and special genetic traits of the Andean Altiplano populations. Am. J. Phys. Anthropol..

[33] Cerrón-Palomino, R. *Lingüística aimara*. **21**, (Centro de Estudios Regionales Andinos ‘Bartolomé de Las Casas’, 2000).

[34] Kircher M, Sawyer S, Meyer M (2012). Double indexing overcomes inaccuracies in multiplex sequencing on the Illumina platform. Nucleic Acids Res..

[35] Maricic T, Whitten M, Pääbo S (2010). Multiplexed DNA Sequence Capture of Mitochondrial Genomes Using PCR Products. PLoS One.

[36] Renaud G, Kircher M, Stenzel U, Kelso J (2013). freeIbis: an efficient basecaller with calibrated quality scores for Illumina sequencers. Bioinformatics.

[37] Renaud G, Stenzel U, Kelso J (2014). LeeHom: Adaptor trimming and merging for Illumina sequencing reads. Nucleic Acids Res..

[38] Renaud G, Stenzel U, Maricic T, Wiebe V, Kelso J (2015). DeML: Robust demultiplexing of Illumina sequences using a likelihood-based approach. Bioinformatics.

[39] Behar DM (2012). A ‘copernican’ reassessment of the human mitochondrial DNA tree from its root. Am. J. Hum. Genet..

[40] Katoh K, Standley DM (2013). MAFFT multiple sequence alignment software version 7: improvements in performance and usability. Mol. Biol. Evol..

[41] Vianello D (2013). HAPLOFIND: A new method for high-throughput mtDNA haplogroup assignment. Hum. Mutat..

[42] Weissensteiner H (2016). HaploGrep 2: mitochondrial haplogroup classification in the era of high-throughput sequencing. Nucleic Acids Res..

[43] Achilli A (2013). Reconciling migration models to the Americas with the variation of North American native mitogenomes. Proc. Natl. Acad. Sci. USA.

[44] Achilli A (2008). Thephylogeny of the four pan-American MtDNA haplogroups: Implications for evolutionary and disease studies. PLoS One.

[45] Bodner M (2012). Rapid coastal spread of First Americans: Novel insights from South America’s Southern Cone mitochondrial genomes. Genome Res..

[46] de Saint Pierre M (2012). Arrival of Paleo-Indians to the Southern Cone of South America: New Clues from Mitogenomes. PLoS One.

[47] Fagundes, N. J. R., Kanitz, R. & Bonatto, S. L. A reevaluation of the Native American mtDNA genome diversity and its bearing on the models of early colonization of Beringia. *PLoS One* **3** (2008). 10.1371/journal.pone.0003157 PMC2527677 18797501

[48] Gómez-Carballa A (2012). A melting pot of multicontinental mtDNA lineages in admixed Venezuelans. Am. J. Phys. Anthropol..

[49] Just, R. S., Diegoli, T. M., Saunier, J. L., Irwin, J. A. & Parsons, T. J. Complete mitochondrial genome sequences for 265 African American and U.S. ‘Hispanic’ individuals. *Forensic Science International: Genetics* **2** (2008). 10.1016/j.fsigen.2007.12.001 19083815

[50] Kumar, S. *et al*. Large scale mitochondrial sequencing in Mexican Americans suggests a reappraisal of Native American origins. *BMC Evol. Biol*. **11** (2011). 10.1186/1471-2148-11-293 PMC3217880 21978175

[51] Lee EJ, Merriwether DA (2015). Identification of Whole Mitochondrial Genomes from Venezuela and Implications on Regional Phylogenies in South America. Hum. Biol..

[52] Perego UA (2009). Distinctive Paleo-Indian Migration Routes from Beringia Marked by Two Rare mtDNA Haplogroups. Curr. Biol..

[53] Perego UA (2010). The initial peopling of the Americas: a growing number of founding mitochondrial genomes from Beringia. Genome Res..

[54] Perego UA (2012). Decrypting the mitochondrial gene pool of modern panamanians. PLoS One.

[55] Geppert M (2011). Hierarchical Y-SNP assay to study the hidden diversity and phylogenetic relationship of native populations in South America. Forensic Sci. Int. Genet..

[56] Geppert M (2015). Identification of new SNPs in native South American populations by resequencing the y chromosome. Forensic Sci. Int. Genet..

[57] Barbieri C (2016). Refining the Y chromosome phylogeny with southern African sequences. Hum. Genet..

[58] Van Oven M, Van Geystelen A, Kayser M, Decorte R, Larmuseau MH (2014). Seeing the wood for the trees: A minimal reference phylogeny for the human Y chromosome. Hum. Mutat..

[59] Mazières S (2008). Uniparental (mtDNA, Y-chromosome) polymorphisms in French Guiana and two related populations - Implications for the region’s colonization. Ann. Hum. Genet..

[60] Purps J (2014). A global analysis of Y-chromosomal haplotype diversity for 23 STR loci. Forensic Sci. Int. Genet..

[61] Di Corcia, T. *et al*. East of the Andes: The genetic profile of the Peruvian Amazon populations. *Am. J. Phys. Anthropol*. 10.1002/ajpa.23209 (2017). 10.1002/ajpa.23209 28343372

[62] R Development Core Team. R: A Language and Environment for Statistical Computing. *R Foundation for Statistical Computing, Vienna, Austria* **0**, http://www.R-project.org/ (2016).

[63] Paradis E (2010). pegas: an R package for population genetics with an integrated–modular approach. Bioinformatics.

[64] Kassambara, A. & Mundt, F. factoextra: Extract and Visualize the Results of Multivariate Data Analyses. Available at https://cran.r-project.org/web/packages/factoextra/index.html (2017).

[65] Venables, W. N. & Ripley, B. D. *MASS: modern applied statistics with S*. (New York: Springer, 2002).

[66] Oksanen, J. *et al*. vegan: Community Ecology Package. R package version 2.0-5. http://cran.r-project.org/web/packages/vegan/index.html (2012).

[67] Paradis E, Claude J, Strimmer K (2004). APE: Analyses of phylogenetics and evolution in R language. Bioinformatics.

[68] Becker, R. A., Wilks, A. R., Brownrigg, R. & Minka, T. P. maps: Draw Geographical Maps, 2013. R package version, 2–3 (2013).

[69] Hijmans, R., Williams, E., Vennes, C. & Hijmans, M. Package ‘geosphere’. Available at: ftp://sunsite2.icm.edu.pl/site/cran/web/packages/geosphere/geosphere.pdf (2015).

[70] Wickham, H. *ggplot2: Elegant Graphics for Data Analysis*. (Springer-Verlag, 2009). 10.1007/978-0-387-98141-3.

[71] Kahle D, Wickham H (2013). ggmap: Spatial Visualization withggplot2. R J..

[72] Drummond AJ, Suchard Ma, Xie D, Rambaut A (2012). Bayesian Phylogenetics with BEAUti and the BEAST 1.7. Mol Biol Evol.

[73] Soares P (2009). Correcting for purifying selection: an improved human mitochondrial molecular clock. Am J Hum Genet.

[74] Darriba D, Taboada GL, Doallo R, Posada D (2012). jModelTest 2: more models, new heuristics and parallel computing. Nat. Methods.

[75] Baele G, Li WLS, Drummond AJ, Suchard MA, Lemey P (2013). Accurate model selection of relaxed molecular clocks in bayesian phylogenetics. Mol. Biol. Evol..

[76] Kass RE, Raftery AE (1995). Bayes Factors. J. Am. Stat. Assoc..

