# Supplementary material for: Enclaves of genetic diversity resisted Inca impacts on population history
Source: Sci Rep. 2017 Dec 12;7:17411. doi: 10.1038/s41598-017-17728-w (PMC5727115; doi:10.1038/s41598-017-17728-w)
Supplement: Supplementary file 1 — Supplementary Material [file 41598_2017_17728_MOESM1_ESM.pdf]

Supplementary Material

Enclaves of genetic diversity resisted Inca impacts on population history

Chiara Barbieri, José R. Sandoval, Jairo Valqui, Aviva Shimelman, Stefan Ziemendorff, Roland Schröder, Maria Geppert, Lutz Roewer, Russell Gray, Mark Stoneking, Ricardo Fujita, Paul Heggarty

Supplementary Text

1. Archaeological, historical and linguistic characterization of Chachapoyas, within the context of northern Peru

Before one can validly assess how genetic data might inform on the demographic history of Chachapoyas within northern Peru, a number of clarifications are in order about the broader historical, archaeological and linguistic contexts through which any genetic results must be interpreted. Human history in this region has played out in a distinctive environment, home to a succession of complex societies up until European conquest.

1.a. Archaeology

In archaeology, the significance of northern Peru lies not least in forming the lowest elevation corridor between Amazonia and the Pacific (the 'Huancabamba deflection', [1]:175-178). This route has long been identified as a preferential gateway for exchange across a huge range of environmental and cultural diversity[2]. It was also the likely route by which several key crops, first domesticated on the fringes of Amazonia, reached the highlands and coasts, and came to underlie the rise of Andean civilization there[3,4].

Peru went on to host a millennial succession of complex societies, culminating in the Inca Empire: vast, but short-lived, under a century in most regions. Before the Incas, the archaeological record reveals a panoply of distinct regional cultures through time. Among them was the Chachapoya culture, spanning roughly five centuries from ~1000 C.E. until the region was conquered by the Inca Empire in 1470[5]. The population identified as Chachapoya

lived in the cloud forest, where the eastern slopes of the Andes begin the environmental transition to Amazonia[6]. Chachapoya served as a collective term for a series of independent political entities connected by a common architecture, art (ceramic style) and iconography[5,7]. “Chachapoyas” remains as the name of a modern-day province within the administrative region named “Amazonas”, but which in fact encompasses highlands still over 3000m, riven by deep valleys. The Chachapoya population left an extensive archaeological record of sarcophagi, characteristic circular-plan houses and massive fortifications, the immediately recognizable landmarks of the region.

1.b. History

The Chachapoya are also extensively mentioned in the earliest historical sources here, i.e. Spanish chronicles that took down oral accounts of Inca rule (largely just from the Inca perspective, however). These report the Chachapoyas’ long resistance to the Incas — ultimately unsuccessful, but not before earning them some renown as the “warriors of the clouds”[6].

So although the lack of a written historical record before the Spanish conquest does generally pose a major limitation on reconstructing past population movements, exchanges and replacements, in the case of Chachapoyas, at least, the chronicles give us rather more coverage to go on. Indeed, Chachapoyas is reported as a clear example of the Inca state policy of forced resettlements (so-called “mitma”), particularly of recalcitrant, rebellious populations. Historians report that "Altogether, about three to five million people out of a total of ten to twelve million resettled in new locales." ([8]: 265). To judge from such reports, Chachapoyas was one of few regions where the local population faced “essentially complete removal”, according to[9] (357, 373), who maps over a dozen locations across the Inca Empire to which Chachapoyas populations were displaced. Balancing resettlements into Chachapoyas are less documented, although given general Inca policies, likely sources would have been other known rebellious regions across the empire, and/or ecologically similar zones also on the Andes/Amazonia transition[10].

On the other hand, this vision of near total replacement rests on a record that is only semi-historical, potentially prone to significant exaggeration, and whose interpretation is often highly controversial ([9]: 91-118). Other sources in fact state that although some local populations were removed, very few people were brought in from other regions of the empire to replace them ([11]: 130-132). That, however, leaves the presence of Quechua here unexplained, if not by movements before or after the Incas.

A genetic perspective is therefore especially valuable to test the claims from early Spanish chronicles and to clarify the origin and the demographic history of the present-day Chachapoya population.

1.c. Language: Chacha and Quechua in northern Peru

The presumed native language of Chachapoya culture (referred to by linguists as "Chacha") is extinct, like all other non-Quechua languages once spoken in the highlands and lowlands of northern Peru. Vestiges of Chacha survive only in present-day placenames, surnames, and in certain phonetic and lexical characteristics of the Spanish spoken in the region today[12–16]. Chacha language presumably likewise left substrate traces in the particular forms of Quechua that came to be spoken in the same territories.

Quechua is the most widely spoken native language family of the Americas, although progressively going extinct in many regions, not least Chachapoyas itself. It is widely known that one particular form of Quechua served as a *lingua franca* for the Inca Empire, but that administrative use alone can explain only a small part of Quechua's current distribution, however. The mere fact that a region speaks Quechua must not be considered superficially: it has no necessary, exclusive association with Inca influence. The match is far from one-to-one. Many regions already spoke Quechua before they were conquered by the Incas, and other regions within their empire appear never to have switched to Quechua. The distribution of the Quechua family reflects many other demographic and cultural processes, both before and after the Incas —i.e. driven by some of the predecessor cultures to the Incas, and also by their Spanish colonial successors[17,18]. The Spanish regime initially promoted the use of Quechua for communication with the native population in many regions. Spanish missionaries purposely introduced Quechua to some regions as the language of choice for evangelising the indigenous population. Colonial records are clear on this only in some regions, however, particularly parts of Amazonia.

Moreover, even where the Incas may indeed have introduced Quechua, by forced population resettlements, they drew the source populations from all over their Empire. So the Quechua spread by the Incas was not necessarily the 'QIIc' variety spoken in their own Cuzco homeland. On the contrary, the *lingua franca* of the Empire was in fact 'Chinchaysuyo Quechua'[19,20]. This was natively spoken not in the Inca capital Cuzco but by populations around Chincha, immediately south of the Lima region on the south-central coast of Peru — or at least that is the general assumption in the linguistic literature, for in fact all direct trace of Quechua in the Chincha region vanished within a few decades of the Spanish conquest.

In the traditional classification of Quechua, it is this past form of Quechua, presumed to have been spoken in Chincha, that is taken as ancestral to all modern forms of QIIb[20]. These

include the Quechua spoken (now by just a last few elderly speakers) in small communities around Chachapoyas. Classed with it in the QIIb clade is the Quechua spoken in another of our sample locations, the town of Lamas, near the city of Tarapoto, ~300 km east of Chachapoyas in the Amazonian lowlands (here represented by the genetic sample from the indigenous neighbourhood known as Wayku). Also classed within QIIb is the 'Inga' spoken in one pocket of southern Colombia, and the much more widely spoken Ecuadoran 'Kichwa', in both the Andean highlands and Amazonian lowlands of Ecuador.

Given the multiple possible explanations for the presence of Quechua in any one region, it is still not fully understood exactly when the language reached Chachapoyas and Lamas. There are at least no strong archaeological candidates for significant enough impacts from any Quechua-speaking region before the Inca period — but that does not exclude Spanish colonial impacts. Nor is there consensus on the extent to which the characteristics of Chachapoyas Quechua reflect those that already defined a clear QIIb branch at the time, or arose independently in Chachapoyas by a linguistic substrate effect.

It is relevant also that in northern Peru, Quechua never seems to have been dominantly established. It was left spoken only scattered across the region, and notably, in significantly diverse forms. Besides Chachapoyas and Lamas, the only other Quechua-speaking communities in the north Peruvian highlands lie further westwards in Cañaris and Incahuasi (Lambayeque region) and around Cajamarca. These are traditionally classified into a separate clade, 'Quechua IIa', although they do share occasional linguistic features with the putative QIIb into which the Chachapoyas and Lamas varieties have been classed. In fact, the very validity of the QIIa and QIIb clades has long been challenged, and there remains uncertainty and controversy as to the origins and exact linguistic relationships that all of these forms of Quechua have to each other, and to the other main branches: 'QI' in central Peru and 'QIIc' in southern Peru, Bolivia, and north-west Argentina[21,22].

To classify certain forms of Quechua all within a putative QIIb branch entails, implicitly, a historical hypothesis: that there once existed a Proto-QIIb language, spoken by some specific, single population, and that all QIIb languages derive from that single source. An alternative hypothesis is possible, however. Indeed there are linguistic doubts as to whether the supposed QIIb varieties actually form a valid clade at all[21,22]. QIIb is defined on very few linguistic criteria, of questionable validity. Cerrón-Palomino ([20]: 239) mentions just two features shared in all QIIb varieties, of which the second is in any case not unique to QIIb, but found also in some varieties classed as QIIa. The first criterion is that the pronunciation distinction between Quechua /k/ and /q/ consonants is lost, since the latter also becomes /k/: so /qiru/ *wood* and /kiru/ *tooth* become indistinguishable, as both /kiru/. The second criterion is that voiceless stop consonants turn into their voiced counterparts (not native to Quechua) when they follow

a nasal consonant /m/, /n/ or /ɲ/: so /inti/ *sun* becomes /indi/, /inka/ becomes /inga/, and so on. Both of these changes are also highly natural, however, and open to arising independently when Quechua is learnt by speakers of other native languages. Relatively few languages make a /k/‿/q/ distinction, whereas many, unlike original Quechua, do have voiced stop consonants, particularly natural after nasals. So the changes found shared across those varieties may simply reflect similar contexts, of Quechua spreading culturally, by being learnt by multiple populations *in situ*, rather than demographically, brought by an incoming population from a single Proto-QIIb source that may never actually have existed. Genetic data from Quechua-speaking populations offer a means to test between these language hypotheses.

2. Genealogical and linguistic characterization of samples

2.a. Geographical Grouping of Samples

A fieldwork expedition was carried out in the provinces of Amazonas and San Martín, collecting samples from various locations, grouped into six geographically coherent sub-regions for the purposes of our analyses. One of these groups, our only sample from the province of San Martín, was **Wayku**, a neighbourhood of the town of Lamas where a variety of Quechua is still spoken, traditionally classified as a separate sub-branch of QIIb to that of Chachapoyas. Within Amazonas province, meanwhile, samples were assigned to one of five groups, by ancestry from Chachapoyas city, Huancas, Luya, La Jalca, or Utcubamba South.

- The **Chachapoyas city** group covers the city itself, and villages immediately to its north.
- **Huancas** is a village also close to Chachapoyas, but distinguished from it in our analyses in order to explore the village's putative ancestry in populations who migrated from central Peru (including the modern city of Huancayo) before the Spanish conquest[23].
- The **Luya** group covers villages in the province of Luya, to the west of the Utcubamba.
- The **La Jalca** group is centred on the town of the same name, to the east of the Utcubamba river.
- The **Utcubamba South** group covers villages close to the towns of Tingo and Yerbabuena, and further south along the Utcubamba.

2.b. Sampling Policy: Surname Analysis

Sampling was conducted specifically to target individuals with local Chacha surnames, with the aim of reaching descendants of local populations rather than recent migrants there. These surnames were identified from the list first reported by Zevallos Quiñones[14], based on documents dating back to the 16th century, and by tracking down surnames reported in the

earliest civil population records of the municipalities concerned. Surnames were screened for any phonetic or etymological traces that could be linked to either a Chacha or a Quechua linguistic substrate. Indications of Chacha substrate were also based on local placenames, which can typically remain stable even through population movements. The surname report given here focuses on paternal surnames of male and female participants (Supplementary Table 1). The latter are of course not included in the Y-chromosome analysis, and the numbers reported therefore differ slightly from the Y-chromosome dataset. In Chachapoyas province we identified 22 individuals with surnames of possible Chacha origin, making up 21% of the sample. Most of these surnames were already present in the report by Taylor[13]. The individuals are distributed as follows: 1 individual in Chachapoyas city, 1 in Huancas, 5 in La Jalca, 12 in Luya, 3 in Utcubamba South. Thirty-one surnames were identified as typically Quechua, making up 30% of the sample. In the province of San Martín, 11 out of 16 individuals had typical local surnames, but ultimately of putative Amazonian linguistic origin. For both provinces, all remaining individuals had surnames of Spanish origin. The list of individual surnames is not provided, to guarantee anonymity.

The surname origin proportions do not correspond exactly to those of Native American vs. European genetic components, however, at least for the uniparental markers considered: 98% of the maternal and 72% of the paternal lineages belong to a native American haplogroup. The highest Native American ancestry is found in Luya, where it reaches 80% in the paternal line.

We researched the regional archives from 1560 to 1700 and found no fixed practice in surname inheritance. Surnames could be received from either the father or mother, or from neither, although in mixed native/Spanish families, only the Spanish surname was retained. In line with this inconsistent inheritance, the Chacha or Quechua surnames in our samples do not correspond to specific Y-chromosome branches in the network (Supplementary Fig. 5). Nevertheless, Chacha and Quechua surnames are found in branches close to each other, and often correspond to individuals from Quechua-speaking families, particularly in Luya and La Jalca. There is a signal of local ancestry, then, but it conflates the Chachapoya and Inca/Quechua components. Indeed, it is possible that when the Spanish colonial administration first established population registers and required surnames to be assigned, the local indigenous population simply chose their preferred surnames, whether Chacha, Quechua or even Spanish.

2.c. Sampling Policy: Quechua Speakers

We conducted a linguistic survey to assess whether Quechua was (or still is) spoken in each participant's family. In Wayku, Quechua is still actively spoken, although declining in usage

among children. All participants from Wayku either speak Quechua themselves, or have a family member who does. As for the Chachapoyas region, we assume that any Quechua reported among our participants is that characteristic of Chachapoyas (traditionally also classified as part of the putative Quechua IIb clade, but a separate sub-branch of it to Lamas Quechua). On some occasions we were able to confirm this by hearing the participant (or a member of his/her family) speaking, but cannot in all cases rule out that family members may in fact have spoken other varieties of Quechua, learnt elsewhere in Peru, rather than Chachapoyas Quechua.

Within Chachapoyas, the area where Quechua is most present, spoken either by some participants themselves, or by their parents and/or grandparents, is Luya: of 31 individuals sampled, 15 reported some members of the family speaking fluent Quechua, while 13 reported some member of the family speaking at least some Quechua. The villages in Luya province were those where we were able to contact and record the highest number of individuals still speaking Chachapoyas Quechua fluently (five people up to date). A few more speakers are found in villages in the north of Chachapoyas province, but no DNA collection was performed there. In La Jalca, Quechua was also spoken by members of the participants' families, but as they reported, more by their grandparents' generation. Of 19 individuals, 12 reported some Quechua spoken, but not fluently, and five fluently. In the Chachapoyas city group and in Huancas, half of the participants reported Quechua spoken in the family, more fluently in Chachapoyas. Finally, Quechua was rarely present in the family histories of people sampled in the villages in the Utcubamba South group (reported for only 30% of the individuals sampled).

3. Results

3.a. Haplogroup composition

Haplogroup composition varies between our geographical groups (Supplementary Table S2): La Jalca is characterized by a high proportion of haplogroup D (57%), Huancas and Wayku by a high proportion of haplogroup B2 (63 and 60% respectively). All of the D individuals belong to D1 except for one D4h3 individual. One individual assigned to U5a1b was originally from Cajamarca, outside Chachapoyas, and was excluded from the analysis. Another individual from Utcubamba South was assigned to L3e and was similarly excluded from the analysis.

Of the 88 male individuals typed with the basal *SNaPshot*® assay, 66 belong to haplogroup Q. Six are of haplogroup Q-M242, ancestral to Q-M3 (in Luya, Utcubamba_South, and La Jalca) while the other 60 are derived for the most commonly found marker M3. All the Q

samples result ancestral for the 5 downstream positions included in the assay. The sample sizes for Chachapoyas and Huancas are too low to allow for meaningful between population comparisons (see Supplementary Tables S1 and S2 for details). Haplogroup C, also part of the native genetic component in the Americas, is absent from our sample, confirming a scattered presence of this marker in western South America[24].

The non-Q individuals, testifying to the genetic contribution from other continents since European contact, were genotyped as follows. Most individuals were assigned to R-M207, present in 7 individuals in La Jalca, 4 in Luya and 5 in Utcubamba South. The remaining 6 individuals are all derived for FT-M213; three of them are ancestral for KLT-M9 and the other three are derived for the same marker (but ancestral to QR-M45).

3.b. Networks

3.b.1. mtDNA

Supplementary Fig. S2 illustrates the relationships of the mitochondrial genomes in our dataset and sequences available from the literature, divided into the four native haplogroups A, B, C and D. For each of these haplogroups we report new branches, in particular for A, B and D. Mitogenome variation in haplogroup B is not yet well characterized in the population literature for South America, so discovering new lineages is expected. Haplogroups A2 and D1 were already present in the Andean region long before B2, according to aDNA studies[25,26]. For these haplogroups too, we find previously undescribed lineages. We now detail how each of these four haplogroups patterns in our data-set.

- Haplogroup A2 is strongly represented in North and Meso-America[27,28]. Some lineages in our sample form a characteristic separate branch (marked in grey), with representatives from 5 groups: Chachapoyas city, Utcubamba South, Luya, La Jalca and Huancas. A second branch includes one individual from Luya and three from Wayku, while a third branch sets individuals from Utcubamba South, La Jalca and Luya close to sequences from Amazonia[29].

- Haplogroup B2 is generally reported as prevalent in the Andean highlands, in particular the Central and Southern Andes, where it began to diffuse in the last millennium[25,30]. It is also found in pockets of high frequency in some populations of the lowlands, for example in the Mato Grosso and Gran Chaco[31]. In the network (Supplementary Fig. S2b), haplogroup B2 displays two distinct branches: one for individuals from North and Meso-America, and one for individuals from the far south. Meanwhile, the remaining lineages (mostly from Ecuador and Peru[32]) radiate from the centre of the network, without forming distinct branches. Some of the haplotypes in our sample are found in isolate lineages, while in four cases they are found in branches (marked in grey) that bring together individuals from more than one of our geographical sub-groups, three of them sharing branches with other haplotypes from Ecuador.

- Haplogroup C1 has a structure similar to that of haplogroup B, with one clade characteristic of the southern cone. Haplogroup C1 is represented in our sample by isolate lineages only, except for one connection between Luya, Chachapoyas city and Wayku, marked in grey (within lineage C1b2) (Supplementary Fig. S2c).

- Haplogroup D includes the two distinct branches of D1 and D4h3, the latter represented in our sample by just one individual, stemming from an Amazonian cluster. Numerous characteristic lineages in Luya and La Jalca belong to haplogroup D1: their origin again cannot be assigned either to autochthonous Andean lineages or to gene flow from Amazonia, where this haplogroup is at its highest frequency. In supplementary Fig. S2d, three branches are highlighted in grey: two include only individuals from Luya, and one includes individuals from La Jalca together with one individual from Luya.

The relationships between sequences are also visible in the tree generated with BEAST (Fig. 4), where some branches include only individuals of one of our geographical groups. Of two branches within A2, one is found only in Huancas and the other only in Luya. There are also two branches within B2, in Wayku and Huancas, and two sister branches of D4, both in La Jalca.

3.b.2. Y chromosome

Supplementary Fig. S4 displays the network of Y chromosome STR haplotypes (for 23 loci), combining our samples with those from Guevara and colleagues[33]. The broad structure, with regular branching and no clear signals of any recent expansions, is comparable to the network from mtDNA sequences. For the Y chromosome, we confirm the position of the Wayku samples at the tips of divergent branches, without any interaction with the samples from the whole Chachapoyas region. In the network, most of the Wayku and Jívaro samples do show a connection to each other, possibly dating back to remote times (given how long the branches are that separate the Jívaro and Wayku clusters), in line with the hypothesis that both share a common Amazonian origin. The rest of the sample is also structured into separate clusters for each group, each with identical or closely related haplotypes, suggesting a certain degree of population structure (i.e. population differentiation). This effect is particularly visible at the bottom left of the network, where two large clusters of samples from La Jalca and from Luya are found on separate branches. Samples from Huancas are found in a branch close to others from Luya and Chachapoyas, more towards the core than the periphery of these clusters. The haplotype proximity testifies to connections between Luya and Utcubamba South, while La Jalca seems generally more isolated, but with some branches connected to samples from Chachapoyas city. The sample labelled Cajamarca represents people of Cajamarca descent, but in fact sampled in a location geographically closer to our Chachapoyas sample than to the

city of Cajamarca. This “Cajamarca” sample is quite differentiated from all others in our coverage, but does show possible connections to Luya and Utcubamba South. Supplementary Fig. S5 displays the same network after highlighting individuals for their surname characteristics. Individuals with a surname of Chacha or Quechua linguistic origin are found scattered across all major branches except those with mostly Jívaro, Wayku or Cajamarca individuals. Neither Quechua nor Chacha surnames cluster in any genealogical ways. At the top right of the network, two branches present seven individuals with a Chacha surname and no Quechua surnames: these are mostly individuals from Luya and Utcubamba South.

3.c. Haplotype sharing on the regional and continent-wide scales

The haplotype sharing plot (Supplementary Fig. S3) visualizes the similarities between the samples with the full dataset of 23 loci. Data from this study are indicated in bold, and compared to the data published by Guevara and colleagues[33]. The Jívaro, Wayku and Cajamarca samples, more geographically distant from Chachapoyas, do not share any haplotypes with any other populations (only within each population). The two Huancas samples (one taken from[33]) share three distinct haplotypes, across 5 and 11 individuals for each population. The sample that Guevara et al. identify as “Chachapoya”, which covers various villages and has a large sample size of 66, shares haplotypes with all neighbouring groups considered in Fig. S3.

The sharing heatplot (Supplementary Fig. S6), focuses on Chachapoyas, Wayku and the closer parts of the Amazonia and the Andes. This includes populations who speak Quechua IIb from Ecuador and elsewhere in northern Peru. The highland regions covered also include Cajamarca (possibly descendants of speakers of Quechua QIIa), Huancayo (Quechua QI) and Huancavelica (Quechua QIIc), all suggested as sources of ancestry for the Huancas of Chachapoyas[23]. Supplementary Fig. S7 shows a map zoomed in on Northern Peru. Of our five geographical groupings within Chachapoyas, only Utcubamba South shares any haplotypes with populations outside the region, namely identical haplotypes with three groups from Central Peru: the Chopcca from Huancavelica (a highland region, speakers of QIIc); the Yanesha (in the foothills near the transition to Amazonia, speakers of an Arawak language originally from Amazonia, but heavily influenced here by Quechua); and Quechua Huanca (from the highlands around Huancayo, speakers of QI). The Wayku show affinities with Quechua speakers from Loreto province in Amazonia (three distinct haplotypes are shared with Quechua speakers from Andoas), and with other Amazonian populations (Shipibo, Yine, Achuar — marked in green in Supplementary Fig. S7). For our Huancas sample itself, however, we find no direct connection to the samples available from any of the supposed highland migration sources. The Huancas sample is instead more similar to its neighbours within the Chachapoyas region.

When allowing for slight differences between haplotypes (similar haplotypes adjusted for mutation rate), sharing patterns remain the same, in line with a high degree of isolation for our entire population sample from the whole Chachapoyas region (Fig. 5b). The only connections found outside of this region are similar haplotypes that the Chachapoyas city shares with the nearest other Quechua-speakers from Cajamarca and Wayku, with the Shawi and with Quechua-speakers from Cusco, while the Utcubamba South group presents sharing with the Yanesha and with populations from the shores of Lake Titicaca. Our data confirm that the Wayku population is involved in a network of sharing with neighbouring Amazonian populations, and has one long-distance connection to the Palikur (French Guyana).

Within Amazonia, local patterns of sharing are also found, particularly strongly between the Jívaro and Awajún, for example. Ecuadorian populations are not connected with Peruvian ones: the lowland Kichwa-speakers in the database share connections with the Waorani, again challenging any putative connections with their Quechua *linguistic* relatives. The patterns of geographical subgroups are more evident from Fig. S9. The continent is divided into 10 geographical groups. Of these, North Central Peru Andes and the Chachapoyas region share haplotypes within a radius of 100 km; Central Peru Amazonia, North-East Peru Amazonia, and Gran Chaco share within a radius of ~250 km; East Amazonia shares from 250 to ~750 km. Lowland Bolivia and Ecuador display the lowest level of sharing, except for North (Colombia and Venezuela), which displays no sharing at all. Finally, South Central Andes displays the highest amount of sharing, distributed regularly from a radius of a few km up to ~800 km. This continental sharing pattern can be compared to the sharing pattern for five linguistic groups characteristic of the Andes: the four main traditional branches of Quechua and Aymara. Only two populations are assigned as speakers (or possible descendants of speakers) of QI, and likewise only two for QIIa: no sharing is reported for any of these four populations. For QIIb and for Aymara, sharing is found within ~150 km, while QIIc has a long-distance sharing profile comparable to that of the geographical group South Central Andes, described above, with whom they roughly overlap.

Sharing frequency was also correlated with population sample size, to illustrate how exceptional the Chachapoyas case is within our database (Supplementary Fig. S8). For this broad comparison, the samples are summarily divided into either Andean or Amazonian. The figure visualizes how the Chachapoyas city sample shares a small number of haplotypes for the large sample size in our study, when compared to the higher general trend reported in the Andes and the lower general trends in Amazonia. The contrast becomes even starker when sharing between geographically close neighbours is excluded. Figure S8b is restricted to only those pairs of populations more than 40 km apart (the average distance between the populations of the Chachapoyas region). In this case, our target region (with the sole

exception of Utcubamba South) shows the lowest sharing trend, even lower than the general trend in Amazonia. Other populations with smaller (but still valid) sample size and either Amazonian or Andean origin appear along the base line of the plot, with no sharing reported: they are also characterized by genetic distinctiveness, possibly exacerbated by isolation and drift in some cases. For example, Taquile, Parakana, Terena and Awa-Guaja have all very low diversity values, compatible with a scenario of isolation and drift, while Palikur and Tiriyo have higher diversity values, in line with their larger sample sizes.

4. The origin of Chachapoyas populations and microgeographical diversity patterns

Several scenarios have been proposed for the origin of the Chachapoya people (and culture), relying on evidence from archaeology and linguistics. The scenarios can be summarized as follows:

a) An origin in the rainforest, emphasizing cultural connections with the Jívaro, in the lowlands immediately to the north of Chachapoyas[34,35].

b) An origin in the southern highlands of Peru or even Bolivia, assuming a direct connection to populations of the Wari (and/or even Tiwanaku) cultures of the Middle Horizon period (c. 500-1050 AD)[7,36,37].

c) Autochthonous development, emphasizing a degree of continuity in ceramic styles found in the region from the second millennium BCE onwards[5].

d) Diverse, long-distance origins, as a result of Inca resettlement policies. That is, whatever the origin of the pre-Inca Chachapoya population, the Incas largely replaced it — almost entirely according to some sources[9], — with a mixed collection of populations from elsewhere in their Empire.

Our Chachapoyas sample shows a distinct genetic identity, in principle favouring hypothesis (c), an autochthonous development for at least part of the mtDNA and Y chromosome lineages found.

Regarding the Y chromosome in particular, Chachapoyas lacks any haplotypes shared with any other regions, not even with the core Andean exchange network (Fig. 5, Supplementary Figs. S6, S7, S9C), so all other studies of those highland populations would (improbably) have to have missed all surviving descendants of the putative Andean source populations for Chachapoyas. Moreover, our sampling strategy targeting Quechua-speaking regions and participants should if anything bias our sample in favour of populations introduced here by the Incas. This effect argues in particular against hypothesis (d), especially when correlated with a maximum time-frame of 20 generations (~600 years), as suggested by simulations (a

split before this time without migration for an Ne=1000 or less would have likely have recorded some haplotype sharing – see Figure S10). A moderate effect of local population displacement without major replacement, as reconstructed by archaeological sources[11], would seem more realistic according to our genetic data.

Furthermore, the presence of characteristic non-Spanish surnames (linking to local placenames, possibly stable through centuries) and of the Chachapoyas Quechua language is matched by a high proportion of male native ancestry, which provides a link to the pre-Inca substrate. If these lineages have indeed survived from the pre-Inca historical layer, this genetic profile would testify to considerable resilience of the Chachapoya population to Inca occupation, notwithstanding the (questionable) early colonial reports of large-scale forced population movements. This long continuity is difficult to reconcile with hypothesis (b)– although the more distant the time-frame, the more challenging is to trace back a possible population connection. The fast mutation rate associated with the Y chromosome STRs and the low diversity within the only native haplogroup found (haplogroup Q) mean that it is difficult to make inferences on the distant past: comparisons based on pairwise R_{ST} distances do not make it possible to distinguish patterns among Peruvian populations[38].

A direct link with ancient human DNA from Chachapoya remains would represent the only possible evidence to confirm or reject this hypothesis. In this too, this region offers an ideal case-study, since prospects for aDNA recovery are relatively good here. The widespread practice of mummification in the Chachapoya culture has left abundant human remains across a multitude of archaeological sites.

Beside the time-frame limitations, the allochthonous hypothesis (Amazonia or highlands) could also still hold, given the possibility either that published samples do not yet include representatives of the putative origin source, or that the signal of the original source has become too diluted in the present-day genetic composition of Chachapoyas. For hypothesis (a), only on the maternal side, and only in one of our sample locations (La Jalca), do we find an unusually high frequency of haplogroup D1, generally most frequent in Amazonia[31] (Supplementary Fig S1). Nevertheless, at a finer level this relationship is not matched by any shared haplotypes (Supplementary Fig. S2); rather, we find a distinct pocket of D1 lineages particular to La Jalca. (To explore this further, geneticists must first address the overall paucity of comparative mitogenomic data for South America.)

Our results undermine not only the strictly Amazonian or Andean origin hypothesis, but also the frequent depiction of Chachapoyas as a crossroads on the lowest-elevation corridor between Amazonia and the Pacific coast[2]. As a caveat, it should be acknowledged that uniparental markers are only a limited reflection of the full ancestry of the region: genomic

data could be used to detect possible finer degrees of admixture. High-resolution genomic analysis together with more comparative data are needed to further elucidate diversity patterns in the region.

Fine-scale comparisons at a micro-geographic scale highlighted the internal diversity between the five samples from the Chachapoyas region. Luya and La Jalca, in particular, are our two largest samples (36 individuals from Luya and 23 from La Jalca). These populations stand less than 50 km apart (albeit on opposite sides of the main Utcubamba valley), yet they present characteristic lineages, distinguished on separate branches of the Networks for both mtDNA (Supplementary Fig. S2) and the Y chromosome (Supplementary Fig. S5). In mtDNA only, the two samples differ in internal diversity: Luya has higher nucleotide diversity, and La Jalca a high overall Φ_{ST} pairwise distance to all other populations (Table 1). This implies different maternal demographic histories for these populations: La Jalca more isolated, Luya in greater contact with other populations, although exactly when this admixture may have occurred cannot yet be specified. The BSPs (Fig. 3b) also indicate that Luya had the larger population size. Historical reconstructions do not suggest a specific scenario of isolation for La Jalca that would explain the genetic drift experienced. The highest diversity found in Luya could be a sampling effect: the samples for this population were drawn from small villages scattered over a wider geographic range within the province of Luya. The proposed structure between these two populations could also be the legacy of different identities that survived from before the Inca period, between distinct populations that archaeologists bring together under the umbrella of "Chachapoya culture". Overall, the microgeographical differences found between the populations studied, and the new genetic lineages described for both mtDNA and Y chromosome, define an enclave of Native American genetic diversity that merits further, higher-resolution investigation at the genomic level.

Supplementary References:

1. Reynel, C., Pennington, R. & Särkinen, T. *Cómo se formó la diversidad ecológica del Perú*. (2013).
2. Lathrap, D. W. The antiquity and importance of long-distance trade relationships in the moist tropics of pre-Columbian South America. *World Archaeol.* **5,** 170–186 (1973).
3. Piperno, D. & Pearsall, D. *The origins of agriculture in the lowland Neotropics*. (Academic Press, 1998).
4. Pearsall, D. Plant domestication and the shift to agriculture in the Andes. *Handb. South Am. Archaeol.* (2008).
5. Church, W. & Von Hagen, A. in *The Handbook of South American Archaeology* (eds. Silverman, H. & Isbell, W. H.) 903–926 (Springer, 2008).
6. Muscutt, K. *Warriors of the Clouds: A Lost Civilization in the Upper Amazon of Peru. Univ. New Mex. Press, Albuquerque* (University of New Mexico Press, 1998).
7. Bandelier, A. *The Indians and aboriginal ruins near Chachapoyas in northern Peru*. (Historical Records and Studies, 1907).
8. D'Altroy, T. N. in *The Archaeology of Colonial Encounters. Comparative Perspectives* (ed. Stein, G.) 263–295 (School of American Research Press, 2005).
9. D'Altroy, T. N. *The Incas*. (John Wiley & Sons, 2014).
10. Lerche, P. *Los Chachapoya y los Símbolos de Su Historia*. (Ediciones y Servicios Gráficos César Gayoso, 1995).
11. Schjellerup, I. *Incas y españoles en la conquista de los chachapoya*. (Fondo editorial de la Pontificada Universidad Católica del Perú, Instituto Francés de Estudios Andinos, 2005).
12. Torero. Áreas toponímicas e idiomas en la sierra norte peruana. Un trabajo de recuperación lingüística. *Rev. Andin.* 217–248 (1989).
13. Taylor, G. *Estudios lingüísticos sobre Chachapoyas*. *Travaux de l'IFEA* (2000).
14. Zevallos Quiñones, J. Onomástica prehispánica de Chachapoyas. *Leng. y Ciencias* 3–18 (1966).
15. Valqui Culqui, J. & Ziemendorff, M. Vestigios de una lengua originaria en el territorio de la cultura chachapoya. *Letras* **87,** 5–32 (2016).
16. Valqui Culqui, J. Reconstrucción de la lengua chacha mediante un estudio toponímico en el distrito de La Jalca Grande (Chachapoyas-Amazonas). (University of Lima, 2004).
17. Heggarty, P. & Beresford-Jones, D. in *The Encyclopedia of Global Human Migration* (ed. Ness, I.) (Blackwell Publishing Ltd, 2013).
18. Beresford-Jones, D. & Heggarty, P. in *The Encyclopedia of Global Human Migration* (eds. Ness, I. & Bellwood, P.) 410–16 (Blackwell Publishing Ltd, 2013).
19. Cerrón-Palomino, R. El aimara como lengua oficial de los incas. *Boletín Arqueol. PUCP* **0,** 9–21 (2012).
20. Cerrón-Palomino, R. *Lingüística Quechua. 2nd ed.* (Bartolomé de Las Casas, 2003).
21. Landerman, P. *Quechua dialects and their classification*. (U.M.I. Dissertation Services., 1991).
22. Heggarty, P. Linguistics for Archaeologists: Principles, Methods and the Case of the Incas. *Cambridge Archaeol. J.* **17,** 311–340 (2007).

23. Colin, R. El pueblo de Huancas. *Boletín la Soc. Geogr. Lima* **XXI,** 465–470 (1907).

24. Mezzavilla, M., Geppert, M., Tyler-Smith, C., Roewer, L. & Xue, Y. Insights into the origin of rare haplogroup C3* Y chromosomes in South America from high-density autosomal SNP genotyping. *Forensic Sci. Int. Genet.* **15,** 115–20 (2015).

25. Raff, J. A., Bolnick, D. A., Tackney, J. & O'Rourke, D. H. Ancient DNA perspectives on American colonization and population history. *Am. J. Phys. Anthropol.* **146,** 503–514 (2011).

26. Fehren-Schmitz, L. *et al.* A Re-Appraisal of the Early Andean Human Remains from Lauricocha in Peru. *PLoS One* **10,** e0127141 (2015).

27. Achilli, A. *et al.* Reconciling migration models to the Americas with the variation of North American native mitogenomes. *Proc. Natl. Acad. Sci. U. S. A.* **110,** 14308–13 (2013).

28. Perego, U. A. *et al.* Decrypting the mitochondrial gene pool of modern panamanians. *PLoS One* **7,** e38337 (2012).

29. Fagundes, N. J. R., Kanitz, R. & Bonatto, S. L. A reevaluation of the Native American mtDNA genome diversity and its bearing on the models of early colonization of Beringia. *PLoS One* **3,** (2008).

30. Fehren-Schmitz, L. in *Population Dynamics in Prehistory and Early History. New Approaches by Using Stable Isotopes and Genetics* (eds. Kaiser, E., Burger, J. & Schier, W.) 55—73 (Boston: De Gruyter, 2012).

31. Bisso-Machado, R., Bortolini, M. C. & Salzano, F. M. Uniparental genetic markers in South Amerindians. *Genet. Mol. Biol.* **35,** 365–387 (2012).

32. Brandini, S. *et al.* The Paleo-Indian Entry into South America According to Mitogenomes. *Mol. Biol. Evol.* (2017). doi:10.1093/molbev/msx267

33. Guevara, E. K., Palo, J. U., Guillén, S. & Sajantila, A. MtDNA and Y-chromosomal diversity in the Chachapoya, a population from the northeast Peruvian Andes-Amazon divide. *Am. J. Hum. Biol.* **28,** 857–867 (2016).

34. Koschmieder, K. Los orígenes y el desarrollo de la organización socio-política de la cultura Chachapoya: Una mirada desde la Provincia de Luya, Departamento Amazonas, Perú. in *Antes de Orellana Actas del 3er Encuentro Internacional de Arqueología Amazónica* 243–249 (2014).

35. Bueno Mendoza, A. & Cornejo García, M. Arqueología de la cuenca del río Guabayacu. Región San Martín, Perú. *Investig. Soc.* **13,** 15–58 (2009).

36. Kauffmann Doig, F. & Ligabue, G. Los Chachapoya (s): moradores ancestrales de los Andes amazónicos peruanos. *UAP* **1,** (2003).

37. Middendorf, E. W. *Die Einheimischen Sprachen Perus. Band 2: Die Aimara –Sprache*. (F.A.Brockhaus, 1891).

38. Barbieri, C. *et al.* Between Andes and Amazon: The genetic profile of the Arawak-speaking Yanesha. *Am. J. Phys. Anthropol.* **155,** 600–609 (2014).

Supplementary Figures

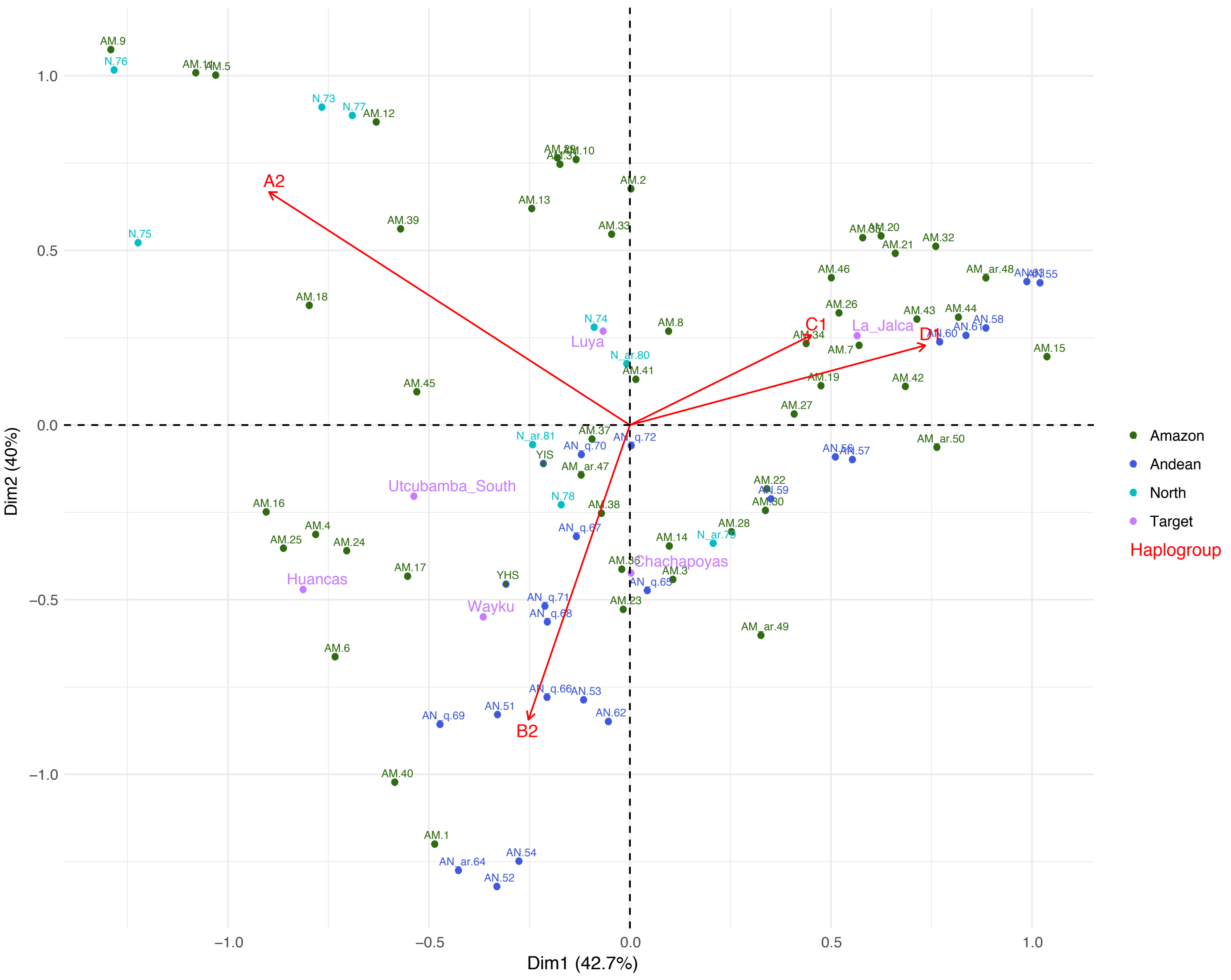

Supplementary Figure S1: CA plot of mtDNA haplogroup frequencies on a continental scale colored for broad geographic range, with target population highlighted. For the corresponding population codes, see Supplementary Table 7 in Barbieri et al., AJPA 2014.

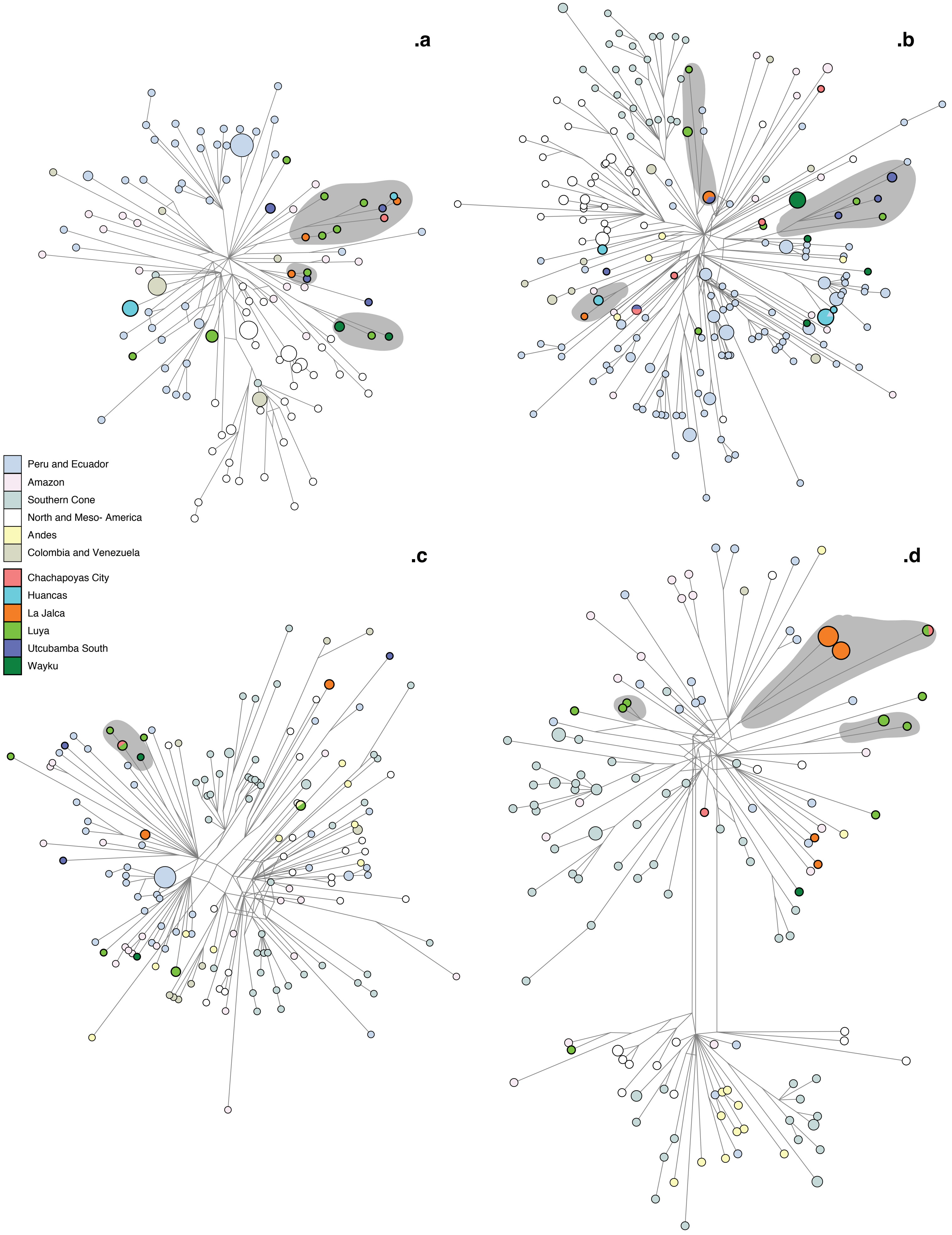

Supplementary Figure S2: Networks on mtDNA genomes: a. haplogroup A, b. haplogroup B, c. haplogroup C, d. haplogroup D. Branches with clusters of population-specific motifs are marked with a gray shadow. Information on the individual sequences is available in Table S4.

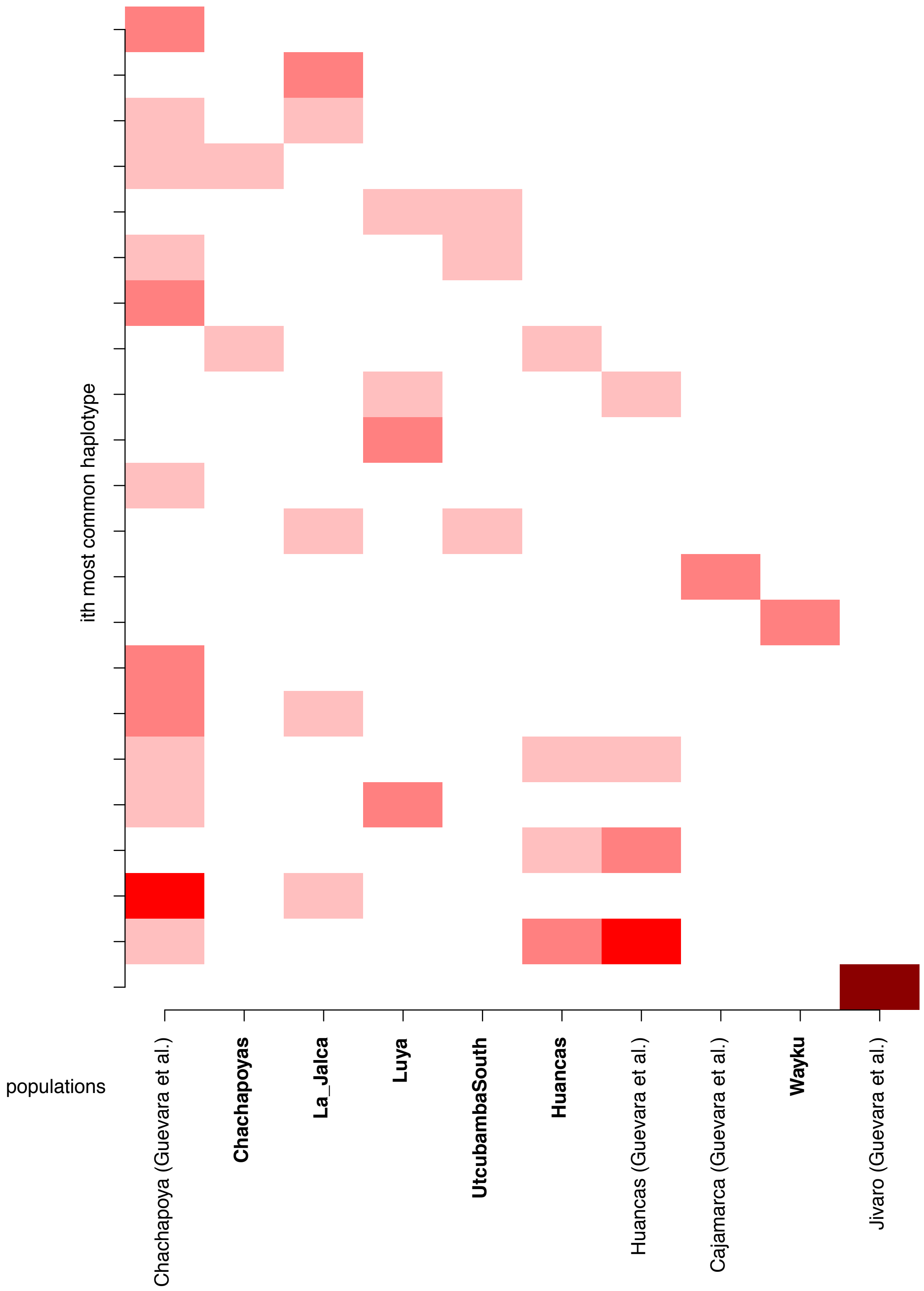

Supplementary Figure S3: Y chromosome haplotype sharing between populations based on the 21 loci STR dataset. Darker colors correspond to a higher number of haplotypes shared.

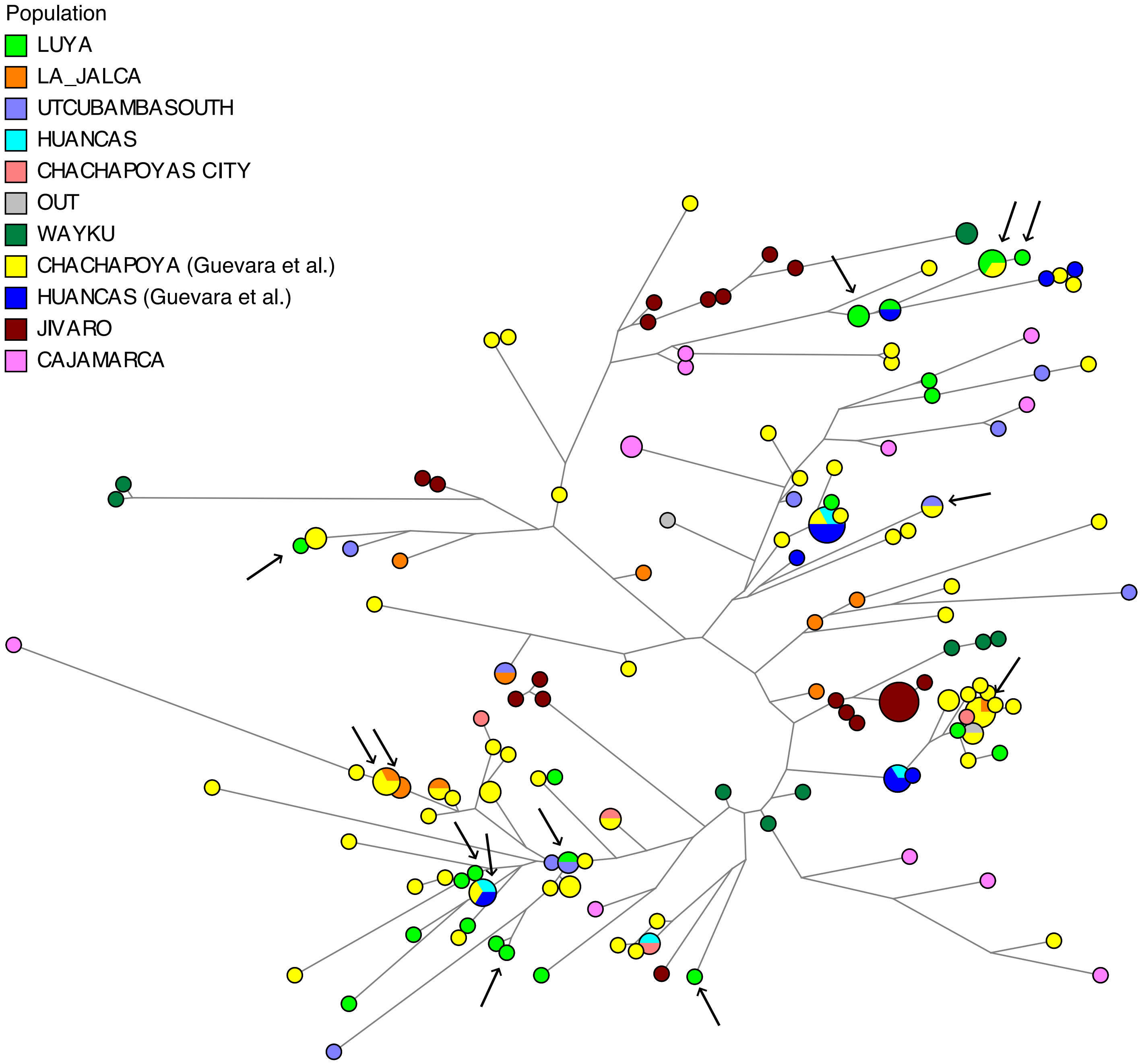

Supplementary Figure S4: Y chromosome median joining network based on the 23 loci STR dataset, colored by population affiliation. The arrows indicate individuals who speak Quechua or who report Quechua to be spoken in their family.

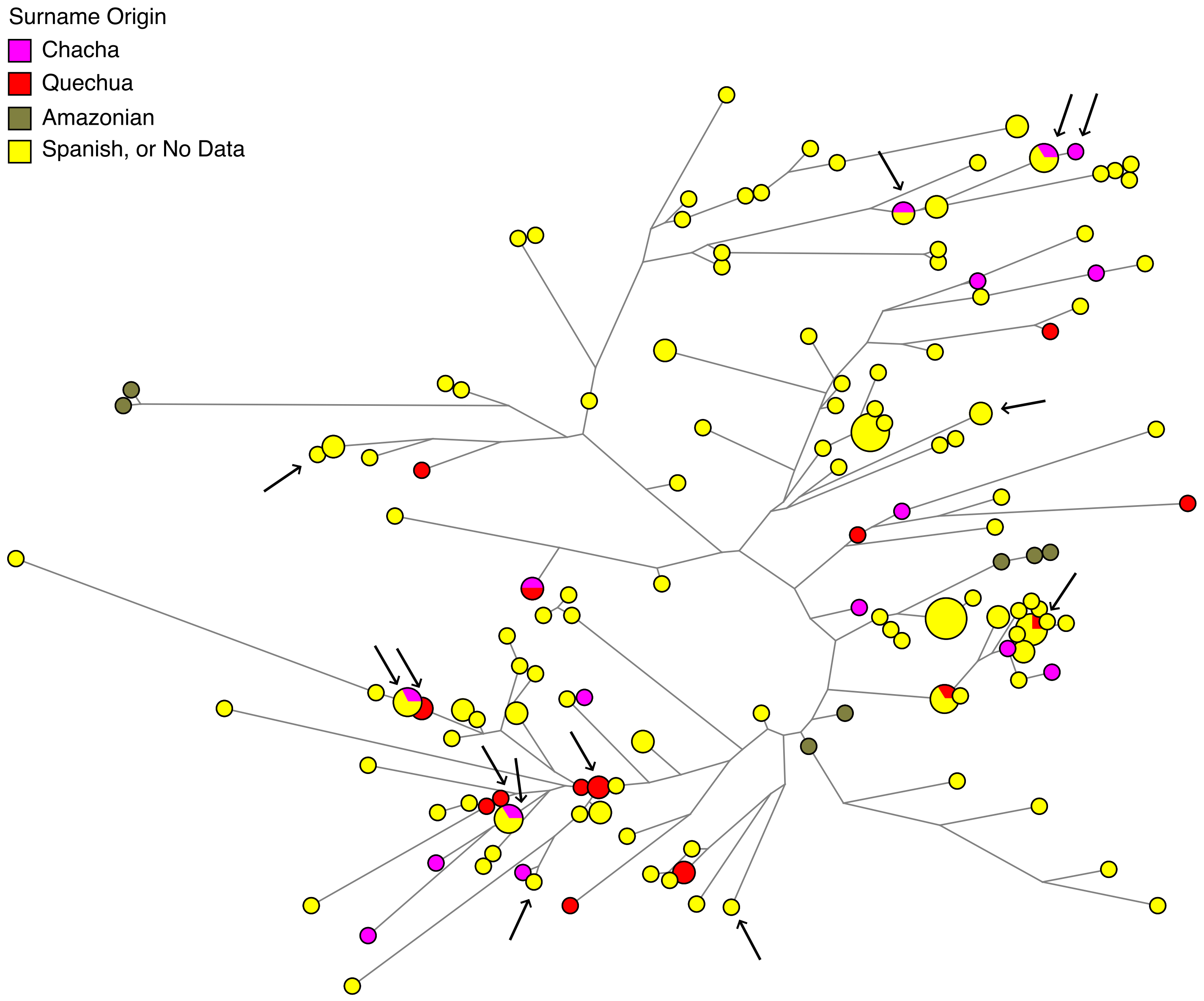

Supplementary Figure S5: Y chromosome median joining network based on the 23 loci STR dataset, colored by presence of surnames of "Chacha" or Quechua origin. The arrows indicate individuals who speak Quechua or who report Quechua to be spoken in their family.

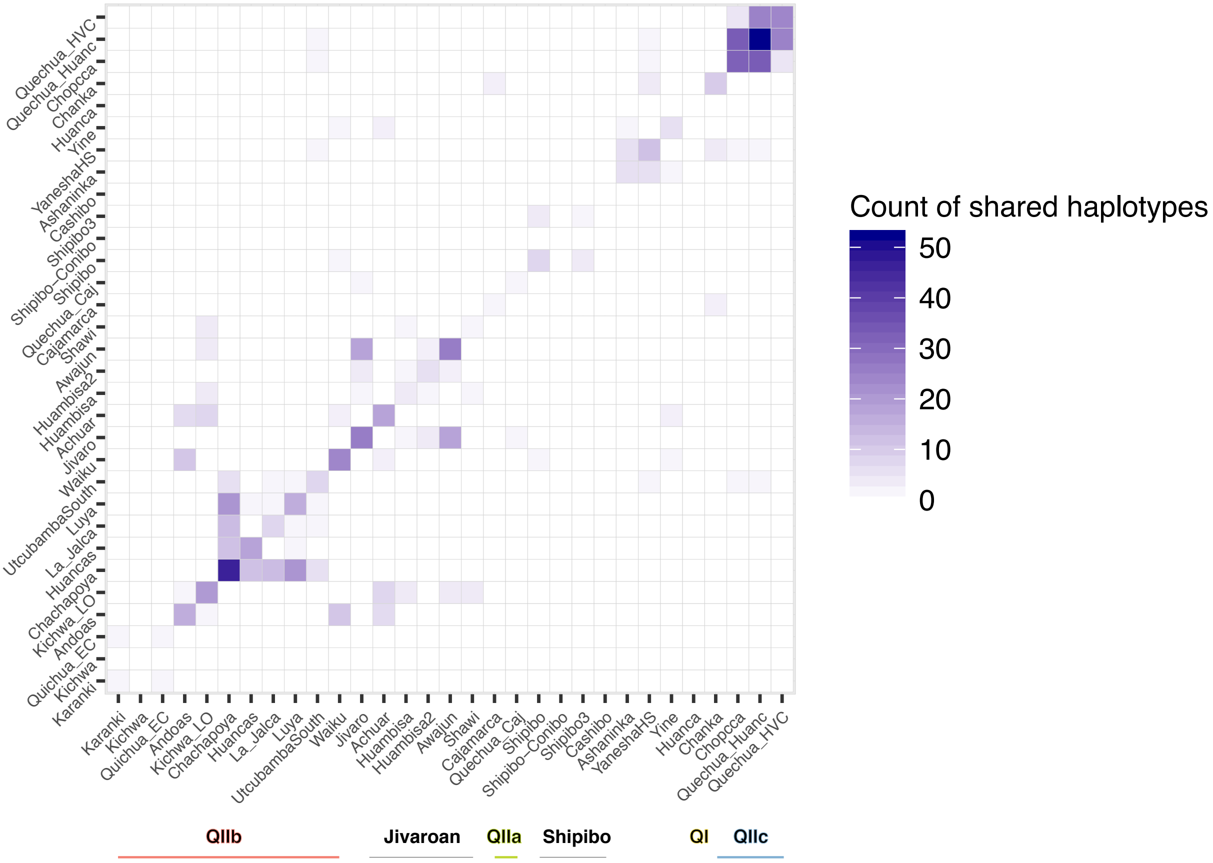

Supplementary Figure S6: Y chromosome haplotype sharing heatplot matrix of populations from northern Peru based on the 17 loci STR dataset. Darker colors correspond to a higher number of haplotypes shared.

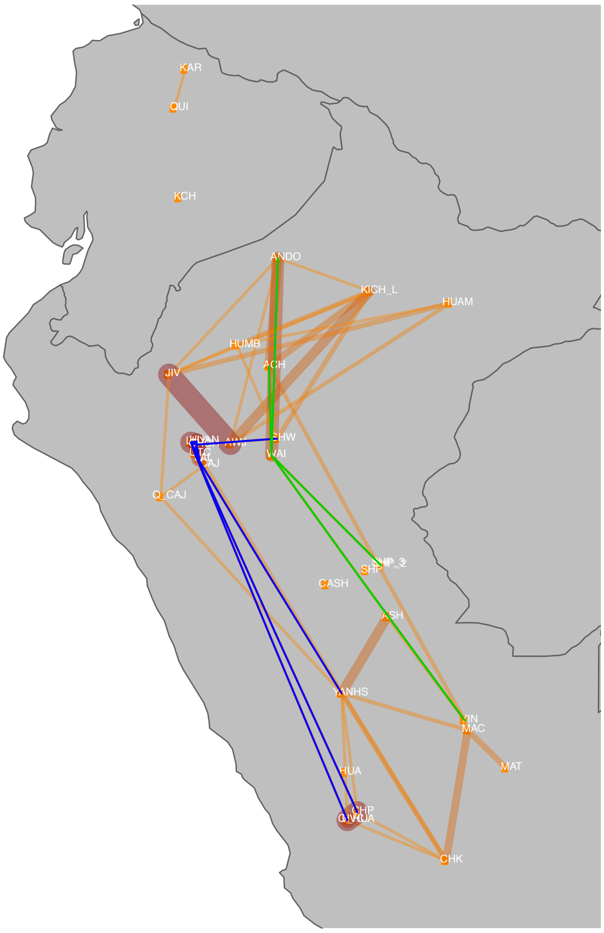

Supplementary Figure S7: Map depicting patterns of Y chromosome haplotype sharing at a local scale, zoomed from Fig. 5. Thin yellow lines correspond to a low exchange frequency, thick red lines correspond to a high exchange frequency. In blue, the network of exchanges which involves the populations from Chachpoyas, Utcumbamba South, Huancas and La Jalca; in green, the network of exchanges which involves the population from Wayku. Map generated in R - version 3.3.0 (www.R-project.org/)

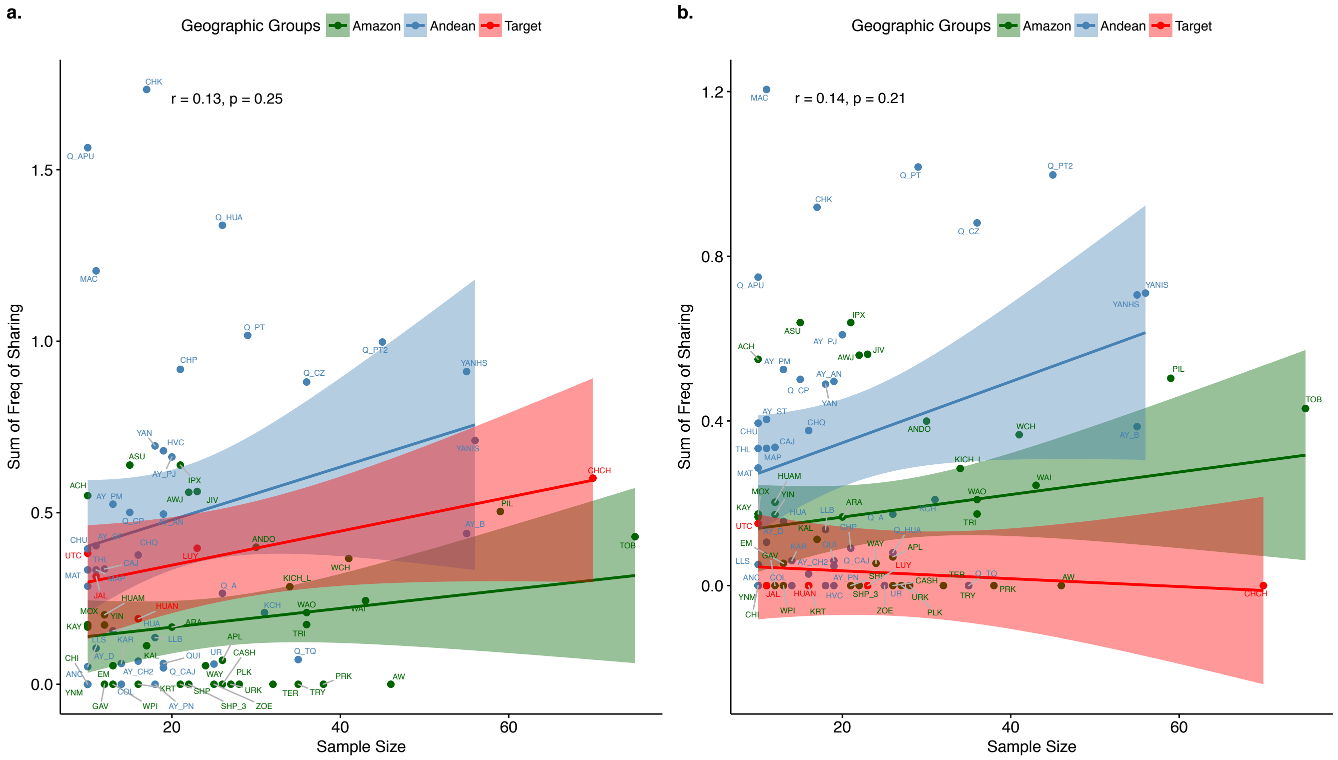

Supplementary Figure S8: Correlation plots between sample size and sum of the frequency of sharing between each population and all the other populations of the dataset, for 81 populations which sample size is equal or larger than 10. **a.** Each sample is compared against all the other 81 populations. **b.** Each sample is compared against populations that are situated at more than 40 km of distance (excluding the sharing occurring between geographic neighbours).

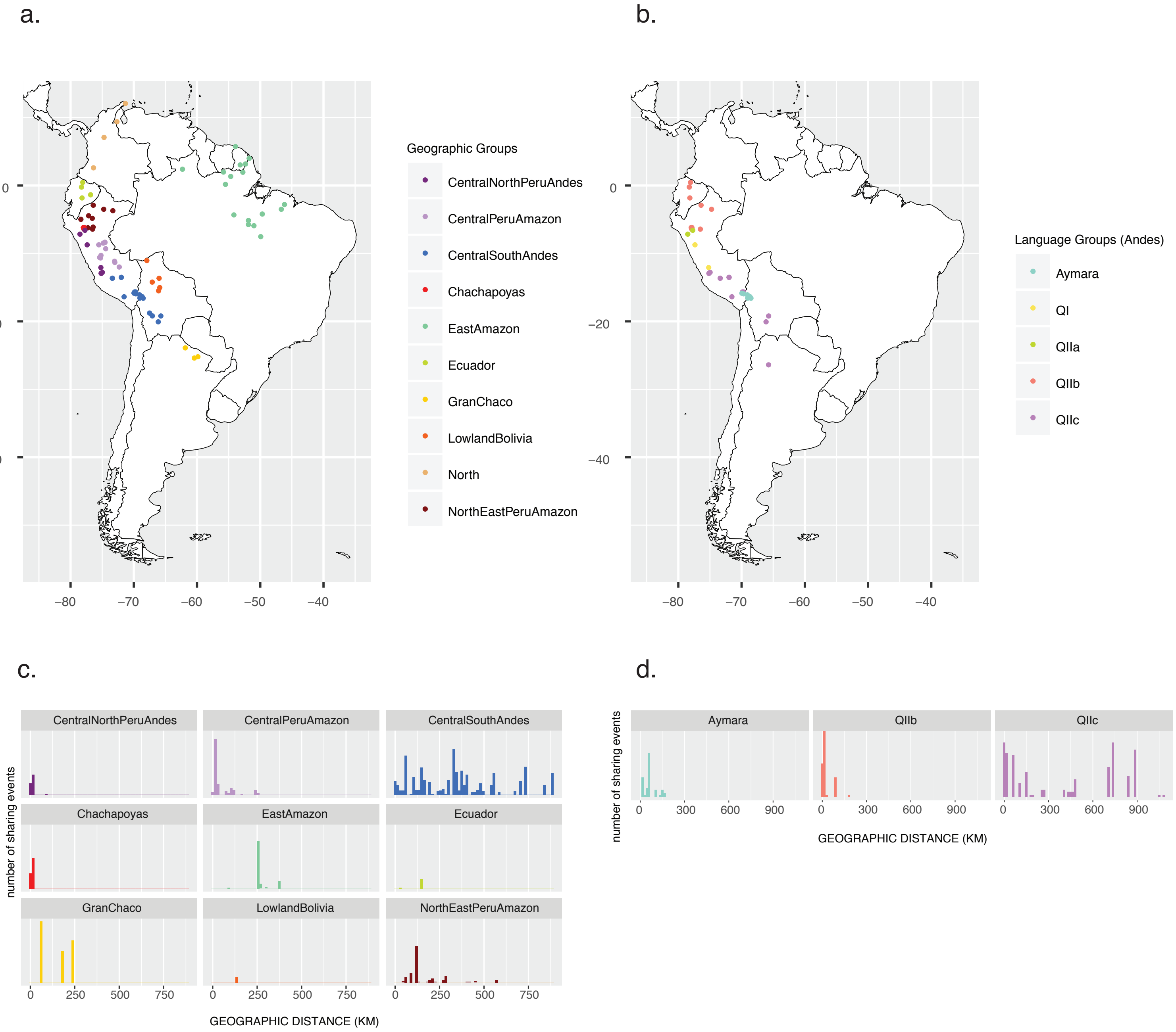

Supplementary Figure S9: Distribution of haplotype sharing events over geographic distances, for geographic and linguistic grouping. a: map of sampling locations for the geographic groups considered. b: map of sampling locations for the Andean linguistic groups considered. c: sharing events per geographic group. Sharing with populations inside and outside the group are considered. Chachapoyas includes Chachapoyas City, Utcubamba South, La Jalca, Luya and Huancas. Populations from the group “North” do not share haplotypes. d: sharing events per linguistic group. Sharing with populations inside and outside the group are considered. Populations from the group “QI” and “QIIa” do not share haplotypes.Map generated in R - version 3.3.0 (www.R-project.org/)

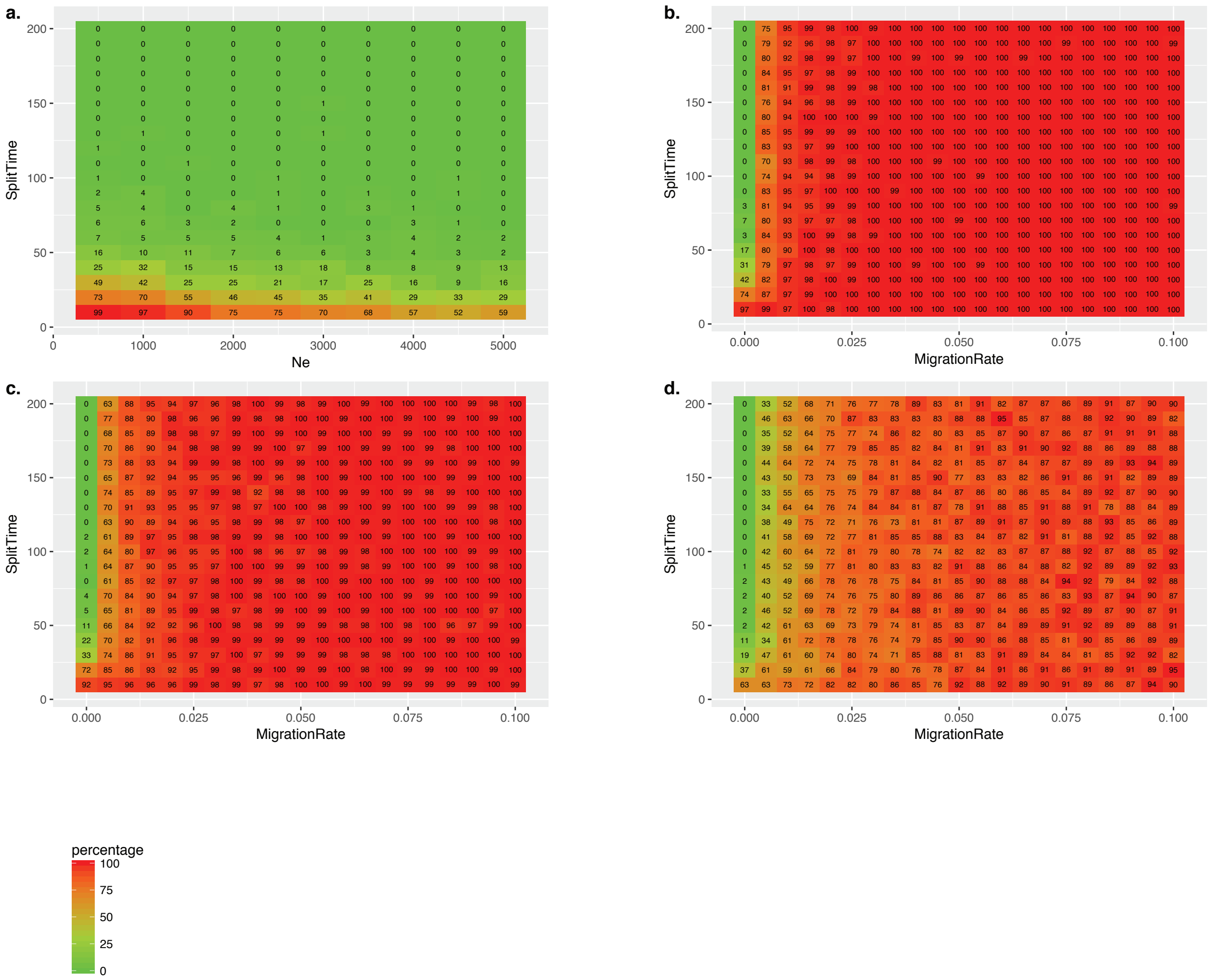

Supplementary Figure S10: Results of the Simcoal simulations for occurrence of haplotype sharing events with variable Ne, Split Time and Migration rate.
a: percentage of simulations where sharing occurs for varying Split Time and Ne (no migration). b: percentage of simulations where sharing occurs for varying Split Time and Migration Rate. Ne is fixed at 500. c: percentage of simulations where sharing occurs for varying Split Time and Migration Rate. Ne is fixed at 1000. d: percentage of simulations where sharing occurs for varying Split Time and Migration Rate. Ne is fixed at 3000.
